# Soft Tissue Radiodensity Asymmetry as Clinical Indicator for Diabetes and Hypertension in Aging

**DOI:** 10.1109/JTEHM.2026.3718689

**Published:** 2026-07-31

**Authors:** Marco Recenti, Alfonso M. Ponsiglione, Carlo Ricciardi, Michela Russo, Kyle J. Edmunds, Francesco Amato, Magnus K. Gislason, Ugo Carraro, Milan Chang, Paolo Gargiulo

**Affiliations:** Institute of Biomedical and Neural EngineeringReykjavik University 64401 102 Reykjavik Iceland; Department of Biomedical ScienceHumanitas University 437807 Pieve Emanuele 20072 Italy; IRCCS Humanitas Research Hospital Rozzano 20089 Italy; Department of Electrical Engineering and Information TechnologyUniversity of Naples Federico II 9307 Naples 80125 Italy; Biomedical DepartmentUniversity of Padova Padua 35131 Italy; Icelandic Gerontological Research InstituteLandspitali University Hospital 37557 101 Reykjavik Iceland; Department of ScienceLandspitali University Hospital 37557 101 Reykjavik Iceland

**Keywords:** Aging, CT-scan, diabetes, hypertension, leg asymmetry, machine learning, radiodensity, soft tissue

## Abstract

Objective: Chronic conditions like diabetes mellitus (DM) and hypertension (HTN) significantly impair physical functioning in older adults, leading to a reduced quality of life and increased risk for disability. This study evaluates the utility of soft tissue radiodensity asymmetry, derived from mid-thigh computed tomography (CT) imaging, as a biomarker for DM and HTN. Method: Using data from the AGES-Reykjavik study, the Nonlinear Trimodal Regression Analysis (NTRA) method was employed to extract 11 geometrical features from fat, muscle, and connective tissue radiodensity distributions derived from cross-sectional CT images of the mid-thigh. Asymmetry indices were calculated as the absolute differences between corresponding left and right leg parameters and were analyzed for their associations with DM and HTN. Results: Statistical analyses demonstrated that DM and HTN status altered the relationship between age and radiodensity asymmetry: DM significantly influenced muscle-specific asymmetry ($\Delta \mu $ musc), while HTN showed directional trends across multiple tissue types that did not survive correction for sex, BMI, and multiple comparisons. Logistic regression identified fat tissue location asymmetry as a key predictor for DM, while connective tissue asymmetry parameters were significantly associated with HTN status. Machine learning models validated these findings, with Random Forest achieving 89.3% accuracy for DM classification and 88.1% for HTN, highlighting the robustness of these asymmetry features. Conclusions: These results establish soft tissue asymmetry as a promising novel biomarker for DM and HTN, reflecting metabolic and vascular dysfunctions. Integrating such measures into clinical practice could enhance predictive models and inform targeted interventions, improving health outcomes for aging populations.

***Clinical and Impact:*** This study presents a novel imaging-based approach for the early identification of diabetes and hypertension in older adults using asymmetry-based digital biomarkers derived from routine CT scans through Non-linear Trimodal Regression Analysis. By capturing bilateral differences in muscle and soft tissue composition and integrating them within explainable machine learning models the proposed framework enables accurate and clinically interpretable classification of metabolic and vascular dysfunctions. Grounded in validated population-level imaging data (AGES-Reykjavík) and designed for seamless integration into existing CT workflows the method can automatically extract features from standard images without additional radiation exposure or patient burden supporting real-time risk stratification during routine radiological assessment. This approach enables early identification of at-risk individuals and potential referral to preventive care pathways while its scalability and compatibility with longitudinal imaging also support patient monitoring over time positioning it as a practical tool for preventive and personalized medicine in geriatric care.

## I. Introduction

 decades, advancements in healthcare science and technology have significantly improved the quality and longevity of life worldwide [1]. However, the rapidly increasing number of older individuals presents a significant challenge for healthcare systems globally [2]. This demographic shift contributes to an economic burden due to the increased need for long-term care, functional decline, and heightened susceptibility to chronic diseases [3], [4].

Among the most prevalent chronic diseases in aging populations are diabetes mellitus (DM) and hypertension (HTN), which are leading contributors to morbidity and mortality in older adults. Approximately 540 million people worldwide (10.5% of the global population) currently suffer from DM, with this number projected to rise to nearly 800 million (12.2%) by 2045 [5]. Among individuals aged 65 years and older, the prevalence of DM approaches 30% [6]. Similarly, HTN affects nearly 70% of individuals over the age of 65, making it one of the most widespread chronic conditions in this demographic [7].

Sarcopenia, the progressive degeneration of skeletal muscle associated with aging, is a condition characterized by decline in both the size and quality of skeletal muscle tissue [8]. This degeneration is frequently accompanied by increased infiltration of non-contractile tissues such as adipose and loose connective tissue. Sarcopenia, DM, and HTN share overlapping pathophysiological mechanisms, including chronic inflammation, insulin resistance, and vascular dysfunction, which together exacerbate the risk of disability and mortality [9].

Older adults with sarcopenia are at an elevated risk of developing DM, while the presence of DM accelerates muscle atrophy and functional decline through mechanisms such as insulin resistance and systemic inflammation [10], [11]. Similarly, HTN compromises muscle perfusion and oxygen delivery, contributing to the prevalence of sarcopenia in individuals with elevated blood pressure [12]. Although sarcopenia does not directly cause HTN, its presence increases the likelihood of HTN occurrence through reduced physical activity and increased arterial stiffness [13]. Notably, individuals with both DM and HTN exhibit a compounded risk of sarcopenia compared to those with either condition alone [14]. These associations underscore the importance of early diagnosis and management of chronic diseases to preserve muscle health and overall functional capacity in aging populations.

Asymmetry in muscle volume and quality between the lower limbs is an emerging area of interest in aging research. Such asymmetry has been linked to impaired physical performance, balance deficits, and increased fall risk [15], [16]. For instance, leg muscle and fat imbalances are associated with reduced gait speed, muscle strength, and flexibility [17], [18]. DM has been implicated in generalized muscle atrophy and strength loss [19], [20], yet its relationship to asymmetry in muscle or other soft tissues remains underexplored. Similarly, while HTN has been linked to muscle weakness and arterial stiffness [21], there is limited evidence addressing the role of soft tissue asymmetry in HTN.

Computed tomography (CT) imaging is a well-established modality for assessing muscle quality and changes associated with sarcopenia [22]. Radiodensity values measured in Hounsfield Units (HU) provide detailed insights into the composition of muscle, fat, and connective tissue [23]. Our research group previously developed the Non linear Trimodal Regression Analysis (NTRA) method, which models the HU distributions of fat, muscle, and connective tissue from mid-thigh CT cross-sections [24]. This approach, first introduced in 2016, yields 11 parameters describing tissue-specific radiodensity distributions, including metrics of amplitude, location, width, and skewness for each tissue type [24], [25]. These parameters have demonstrated high efficiency in predicting BMI, leg strength, and cardiac pathophysiology using machine learning (ML) approaches [27], [28], [29], [30]. For example, tree-based ML algorithms have achieved over 95% accuracy in classifying DM and HTN using single-leg NTRA parameters, and longitudinal models have demonstrated high accuracy in predicting DM incidence over five years [31]. Recent research has expanded this approach by introducing NTRA-derived asymmetry parameters, which quantify differences in soft tissue radiodensity between the left and right legs. These asymmetry parameters have been linked to age-related declines in physical function, such as slower gait speed and longer times to complete functional mobility tasks [32]. However, the potential of these asymmetry measures to serve as biomarkers for chronic conditions like DM and HTN remains unexplored.

This study aims to investigate NTRA-derived asymmetry parameters as predictive features for DM and HTN using a combination of ML and statistical approaches as depicted in Fig. 1. By analyzing these associations between soft tissue asymmetry and chronic disease status, this work contributes to a deeper understanding of the complex relationships among skeletal muscle, metabolic, and vascular health. Importantly, the proposed approach is not intended as a replacement for established diagnostic tools for DM or HTN, but rather as a complementary, opportunistic risk stratification method. By extracting quantitative tissue asymmetry information from CT scans already acquired for other clinical indications, this framework could identify older adults who warrant further metabolic or vascular screening, at no additional cost or radiation burden.

**FIGURE 1.**
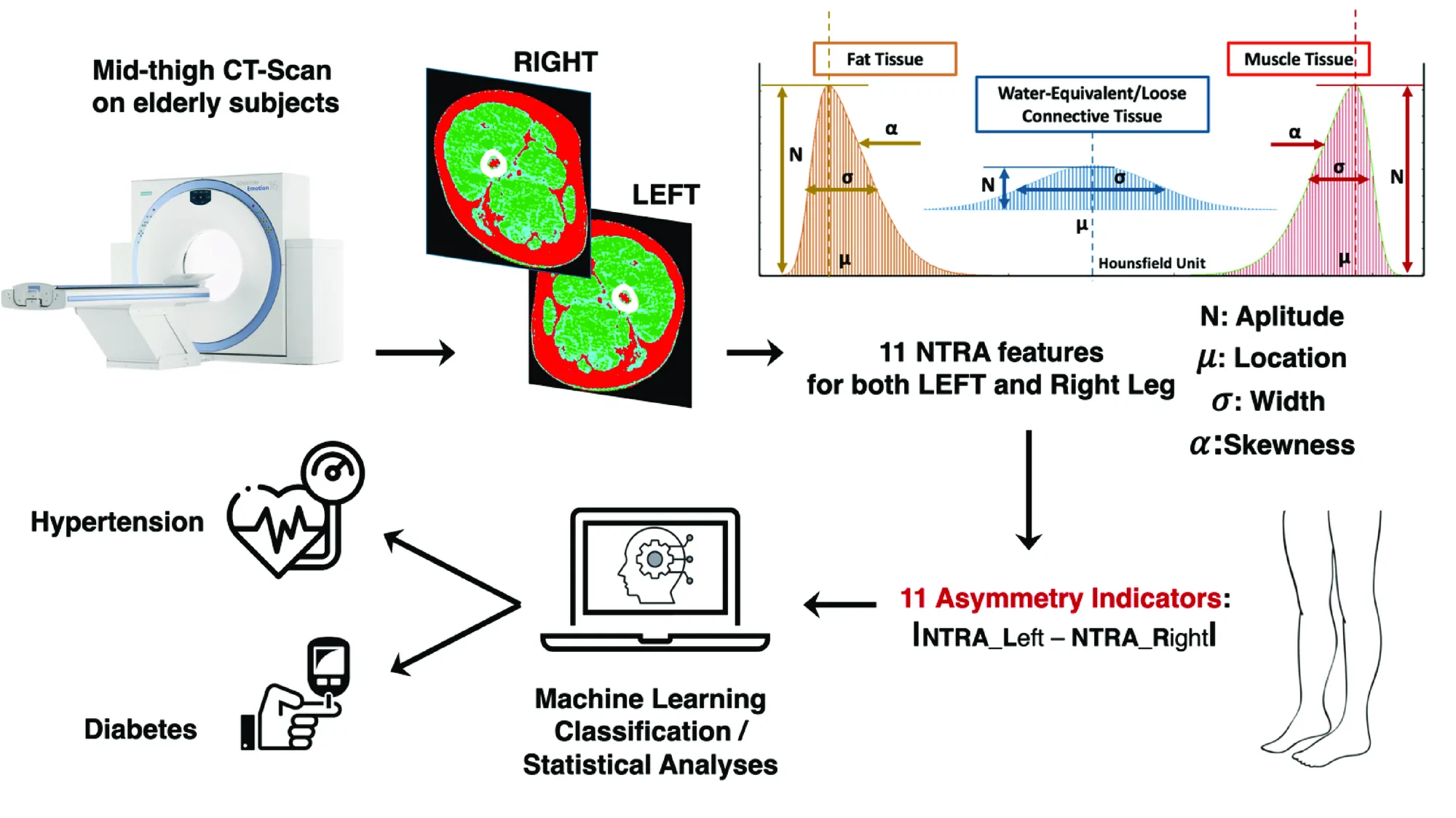
Workflow of the study: after a CT-Scan of both left and right leg the 11 radiodensity distribution NTRA features are extracted from each leg and the 11 asymmetry indicators related to fat, muscle and water equivalent connective tissue are computed. Classification machine learning models are then computed to classify hypertension and diabetes incidence using the data from then elderly subjects-based Icelandic AGES-Reykjavik Dataset.

## II. Methods and Procedures

### A. Participants and AGES-Reykjavik Database

The AGES-Reykjavik dataset [32] comprises two sections, AGES-I and AGES-II, where the same elderly participants underwent multimeric measurements approximately five years apart. This study utilizes data from the first timepoint (AGES-I).

All participants underwent a mid-thigh CT scan and were assessed for the presence of DM and HTN, alongside other comorbidities not addressed in this paper. Participants with diagnosed DM were assigned a binary indicator for its presence, whereas HTN was categorized into three classes: no HTN (class 0), pre-HTN (class 1, defined as systolic blood pressure of 120–139 mm Hg or diastolic blood pressure of 80–89 mm Hg), and HTN (class 2). The three-class HTN categorization was adopted because it reflects the physician-assigned classification recorded in the AGES-Reykjavik dataset, where pre-HTN represents a clinically recognized intermediate risk state. Given the aging nature of the cohort, a high prevalence of subjects with HTN (class 2: 78.9%) was expected and is consistent with epidemiological data in this age group.

### B. CT-Imaging

In the AGES-Reykjavik study, participants underwent bilateral leg imaging using a 4-row CT scanner (Sensation, Siemens Medical Systems, Erlangen, Germany). The scanning protocol targeted the region between the iliac crest and the knee. To ensure accurate localization, the maximum femoral length was measured on an anterior-posterior localizer image, and the scan was centered along the femoral long axis. A single 10-mm-thick cross-sectional slice was then obtained at the midpoint of the thigh, defined as the halfway point between the acetabulum of the hip and the knee joint.

Radiodensity distributions, measured in Hounsfield units (HU), were extracted from each cross-sectional image and analyzed using the NTRA method. The HU spectrum ranged from -200 to 200, with segmented volumes classified into three soft tissue categories: adipose tissue (-200 to -10 HU), water-equivalent and loose connective tissue (-9 to 40 HU), and lean muscle tissue (41 to 200 HU).

### C. Non Linear Trimodal Regression Analysis

The NTRA method considers each HU distribution as a quasi-probability density function by summing two skewed ($\boldsymbol {\alpha \ne 0}$) and one standard Gaussian probability density function ($\boldsymbol {\varphi }$):

\begin{align*} & \sum \limits _{\boldsymbol {i}=\boldsymbol {1}}^{\boldsymbol {3}} \boldsymbol {\varphi }\left ({ {\boldsymbol {x}}, {\boldsymbol {N}}_{\boldsymbol {i}}, {\boldsymbol {\mu }}_{\boldsymbol {i}}, {\boldsymbol {\sigma }}_{\boldsymbol {i}}, {\boldsymbol {\alpha }}_{\boldsymbol {i}}}\right) \\ & =\sum \nolimits _{\boldsymbol {1}}^{\boldsymbol {3}} \frac {{\boldsymbol {N}}_{\boldsymbol {i}}}{{\boldsymbol {\sigma }}_{\boldsymbol {i}} \sqrt {{\boldsymbol {2}} {\boldsymbol {\pi }}}} {\boldsymbol {e}}^{-\frac {\left ({ x-\mu _{i}}\right)^{2}}{2 {\boldsymbol {\sigma }}_{\boldsymbol {i}}^{\boldsymbol {2}}}} {\boldsymbol {erfc}}\left ({ \frac {{\boldsymbol {\alpha }}_{\boldsymbol {i}}\left ({ {\boldsymbol {x}}-{\boldsymbol {\mu }}_{\boldsymbol {i}}}\right)}{{\boldsymbol {\sigma }}_{\boldsymbol {i} } \sqrt {\boldsymbol {2}}}}\right) \tag {1}\end{align*}

In the formula (1), the parameters N, *μ *, *σ *, and *α * represent the amplitude, location, width, and skewness of each distribution, respectively. These parameters are iteratively calculated for each bin in the CT data (x). This approach assumes that each HU distribution is trimodal, corresponding to three distinct tissue types with specific HU ranges. The skewed peaks of fat and muscle distributions are characterized by the skewness (*α *), modeled using the complementary error function (erfc), while the connective tissue distribution is modeled as a symmetric Gaussian.

Finally, after this mathematical analysis, a total of 11 parameters are derived per subject: four (N, *μ *, *σ *, and *α *) for fat and muscle, and three (N, *μ *, and *σ *) for the connective tissue [24].

Asymmetry indices are then calculated by taking the absolute differences in these parameters between the left and right legs, resulting in 11 NTRA asymmetry values per subject [31]:

\begin{equation*} \Delta NTRA={\vert NTRA}_{Left}-{NTRA}_{Right}\vert \tag {2}\end{equation*}

Absolute differences were used rather than signed or relative differences: this choice is consistent with prior work [31] and allows direct comparability with previously published asymmetry indices. The potential information content of signed or normalized asymmetry measures represents a direction for future investigation.

### D. Statistical Analysis

Statistical analyses were conducted using SPSS (v. 29) and R Studio (v. 2024.12). As established in previous research [31], the data were not normally distributed, necessitating a non-parametric approach.

Mann-Whitney U tests were first used to compare NTRA asymmetry parameters between subjects with and without DM, while Kruskal-Wallis tests were employed to differentiate the three HTN groups.

Cross-sectional linear regression models were then constructed to evaluate whether the previously reported [31] third-order polynomial associations of age with each NTRA asymmetry index were moderated by DM or HTN status. Generalized linear models and multinomial logistic regression were then used to examine the logistic associations between age and DM or HTN status. Moderation models were subsequently tested to assess whether NTRA asymmetry influenced these logistic age-DM or age-HTN associations.

Sex (coded as a binary covariate: 0 = male, 1 = female) and BMI were included as covariates in all linear and logistic regression models to control for their known confounding effects on soft tissue radiodensity. Their inclusion did not substantially alter the pattern of significant findings, supporting the robustness of the reported associations. To account for multiple comparisons, all p-values were adjusted using the Benjamini-Hochberg (BH) correction, with statistical significance set at *p < 0.05* p < 0.05.

### E. Machine Learning Classification for HTN and DM Status

This study performed ML classification using Python 3.12.4. Five widely used ML algorithms were employed: Random Forests (RF), ADA-Boost (ADA-B), Gradient Boosting (GB), Support Vector Machine (SVM), and K-Nearest Neighbors (KNN) [33], [34], [35]. RF builds multiple decision trees independently with randomized data and feature subsets, combining their outputs via majority voting to reduce variance. ADA-B iteratively improves the model by focusing on misclassified instances in each training round. GB combines RF’s randomization with ADA-B’s error correction, sequentially refining predictions by addressing prior errors. SVM identifies the optimal hyperplane that maximises the margin between classes in a high-dimensional feature space, using a radial basis function kernel to handle non-linear class boundaries. KNN classifies each sample based on the majority class among its k nearest neighbours in feature space, as measured by Euclidean distance.

A stratified 10-fold cross-validation was performed, alongside hyperparameter tuning for all algorithms. Model performance was evaluated using classification metrics, including accuracy, precision, recall, F1-score, and AUC-ROC (with standard deviations for cross-validation). For the three-class HTN classification, weighted metrics were calculated, excluding AUC-ROC.

Due to significant class imbalance in the dataset, Synthetic Minority Over-sampling Technique (SMOTE) was applied to generate synthetic samples for the minority class, balancing class distributions. SMOTE interpolates between minority class instances and their nearest neighbors to mitigate imbalance [36]. SMOTE was applied within the training folds, not only to increase the representation of minority classes, but also to improve the model’s ability to generalize. The tuning of the hyperparameters was performed using grid search with a 10 k-fold cross validation and the final model parameters were selected based on the highest weighted F1-score. Details on optimal selected hyperparameters are available in the Supplementary Material.

Features’ importance analysis was conducted for the best-performing models to identify the relative contributions of each of the 11 NTRA asymmetry parameters, and secondarily to estimate which of the three soft tissues contributed most to comorbidity classification, where algorithmic interpretation was allowed.

For the best-performing models, explainability analysis [37] has been carried out by means of Partial Dependence Plots (PDPs), a model agnostic global method to explain the effect of one or more features on the overall predictive model outcome by considering marginal effects [38], and by SHapley Additive exPlanations (SHAP), a model agnostic local method for correlating the probability of a predicted class to the value of a particular feature by calculating the average of the marginal contribution of a specific feature to the class prediction across all possible permutations of the features [39]. PDPs were derived to explain the input-output relationships of the classification algorithm for each of the *11 Δ * NTRA asymmetry indicators.

It should be noted that the feature axes in PDP and SHAP plots are expressed in the native units of the NTRA asymmetry parameters (Hounsfield units), for which established clinical thresholds do not currently exist. These visualizations should therefore be interpreted as exploratory tools for understanding model behaviour, and not as clinically actionable thresholds.

To summarize The ML pipeline was implemented as follows: (1) feature extraction of the 11 NTRA asymmetry indices; (2) stratified 10-fold cross-validation with SMOTE oversampling applied within the training folds to address class imbalance; (3) hyperparameter optimization via grid search within each training fold based on weighted F1-score; (4) model evaluation on the validation fold; (5) features importance analysis and lastly explainability analysis. All stochastic components used a fixed random seed to ensure full reproducibility.

## III. Results

### A. Participants Characteristics and Univariate Analyses

Participant characteristics (n = 3128) are described in Table 1. The sample was 58.0% female with a mean ± SD age at CT imaging of *74.9 ±  4.8* years; n = 326 (10.4%) had DM while n = 500 (16.0%) had pre-HTN and n = 2467 (78.9%) had HTN.

**TABLE 1:** Participants characteristics.

Variable	Value
Age (years), mean (SD)	74.9 (4.8)
Female, n (%)	1824 (58.0)
BMI, mean (SD)	27.3 (4.2)
DM, n (%)	
0: no 1: yes	2804 (89.6) 326 (10.4%)
HTN, n (%)	
0: normal 1: pre-HTN 2: HTN	163 (5.2) 500 (16.0) 2467 (78.9)

Notes: BMI = Body Mass Index; DM = Diabetes Mellitus; HTN = Hypertension

Mann Whitney tests comparing the asymmetry parameters of patients with and without DM resulted in only two parameters being statistically significant in distinguishing the groups; both were derived from the muscle tissue: ${\Delta N}_{\text {musc}}$ (p = 0.022) and ${\Delta \sigma }_{\text {musc}}$ (p = 0.022).

Univariate Kruskal Wallis tests comparing the asymmetry parameters of patients with no-HTN, pre-HTN and HTN indicated that no parameter was statistically significant in distinguishing the three groups of HTN.

### B. DM/HTN Status Modifies Linear Associations of Age and NTRA Asymmetry

Table 2 shows results from moderation models examining the interactions of age and DM status on NTRA asymmetry parameters. All models were adjusted for sex and BMI as covariates. DM status significantly modified the association of age on one of the 11 NTRA asymmetry parameters: $\Delta \mu _{\text {musc}}$ (BH-adjusted p = 0.002 for the quadratic interaction term). Fig. 2 illustrates this association stratified by DM status, shown both as a cubic polynomial fit (panel A) and as a linear sensitivity analysis (panel B).

**TABLE 2:** DM status modifies linear associations of age and *Δ * NTRA parameters.

Tissue and Variables		Age x DM			Age^2^ x DM		Age^3^ x DM	
		B (SE)	p	B (SE)	p	B (SE)	p
Fat	*Δ * N	+ 0.41 (1.33)	0.991	+ 0.39 (1.06)	0.991	−0.34 (0.49)	0.991
	$\Delta \mu $	−0.17 (0.49)	0.991	−0.56 (0.38)	0.782	+ 0.21 (0.18)	0.895
	$\Delta \sigma $	−0.31 (0.19)	0.782	−0.04 (0.15)	0.991	+ 0.09 (0.07)	0.872
	$\Delta \alpha $	−0.01 (0.08)	0.991	−0.03 (0.06)	0.991	+ 0.01 (0.03)	0.991
Muscle	*Δ * N	+ 0.10 (1.28)	0.991	−0.29 (1.01)	0.991	−0.17 (0.47)	0.991
	$\Delta \mu $	−0.00 (0.23)	0.991	+ 0.74 (0.18)	0.002*	−0.23 (0.09)	0.108
	$\Delta \sigma $	−0.03 (0.19)	0.991	+ 0.03 (0.15)	0.991	−0.00 (0.07)	0.991
	$\Delta \alpha $	+ 0.02 (0.05)	0.991	+ 0.08 (0.04)	0.514	−0.03 (0.02)	0.782
Conn	*Δ * N	+ 0.48 (0.91)	0.991	+ 0.85 (0.72)	0.895	−0.16 (0.34)	0.991
	$\Delta \mu $	−0.35 (0.60)	0.991	+ 0.02 (0.47)	0.991	+ 0.18 (0.22)	0.991
	$\Delta \sigma $	+ 0.10 (0.89)	0.991	+ 0.44 (0.70)	0.991	−0.21 (0.33)	0.991

Notes: DM = diabetes mellitus; HTN = hypertension; NTRA = nonlinear trimodal regression analysis; *N *= amplitude; *μ *= location; *σ *= width; *α *= skewness; all models include for sex and BMI as covariates.

**FIGURE 2.**
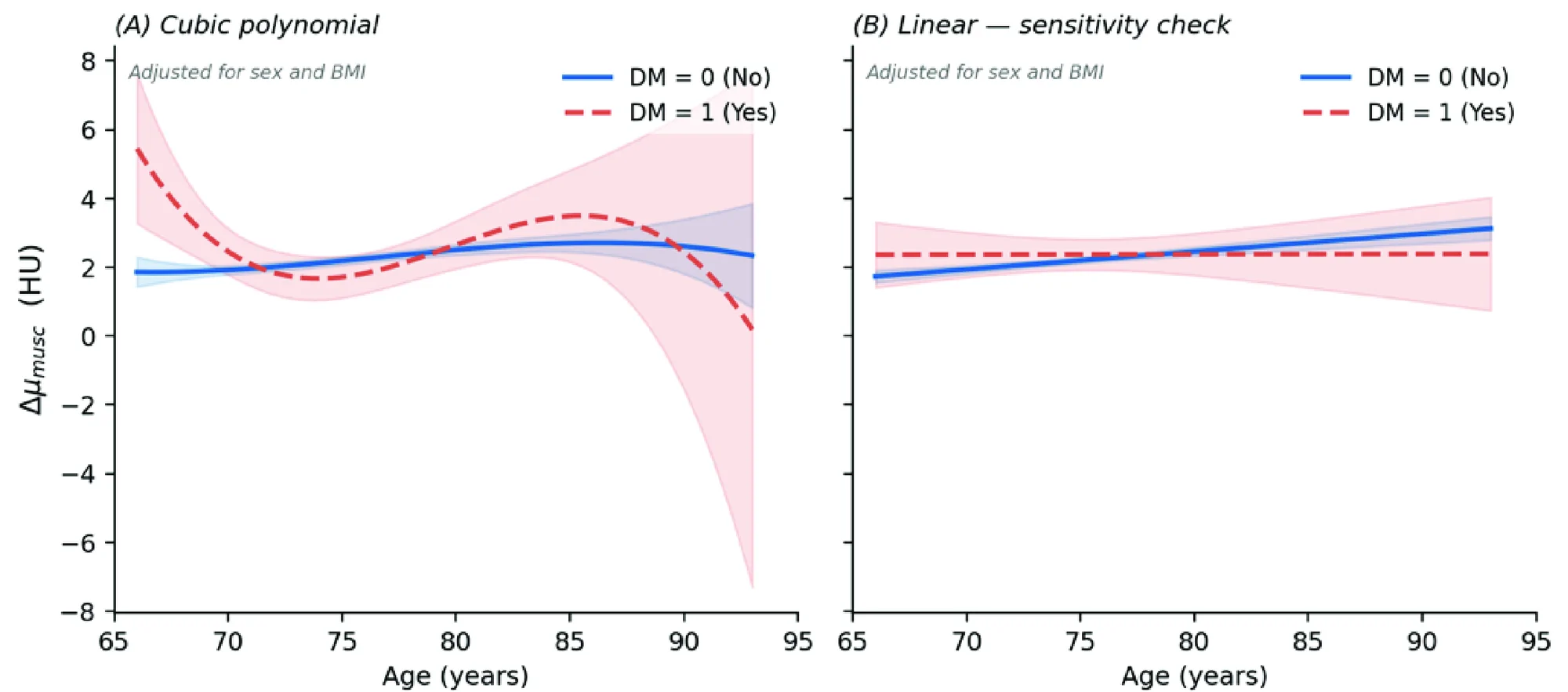
Association between age and stratified by DM status. A third-order polynomial fit (solid lines) (A) and a linear fit (B) adjusted for sex and BMI with 95% confidence intervals (shaded regions) is shown for both DM groups: DM = 0 (no, blue), DM = 1 (yes, red).

Table 3 shows results from moderation models for HTN status. After adjustment for sex and BMI and application of BH correction, no interaction term reached statistical significance. This pattern contrasts with the unadjusted results and reflects the more conservative threshold imposed by covariate adjustment combined with BH correction across 33 simultaneous tests. The directional trends observed in the data, particularly for fat and muscle asymmetry parameters, remain consistent with those reported in prior work [30], [31] and are illustrated in Fig. 3. It illustrates the age–asymmetry associations stratified by HTN status. While each association varied in polynomial shape and direction, asymmetry generally increased with age, with the exception of $\Delta \mu _{\text {conn}}$, which decreased. Sensitivity analyses using linear fits are shown alongside the cubic models in Fig. 3 and Fig. 4 (sensitivity panels); the directional consistency across model orders supports the robustness of the reported trends.

**TABLE 3:**
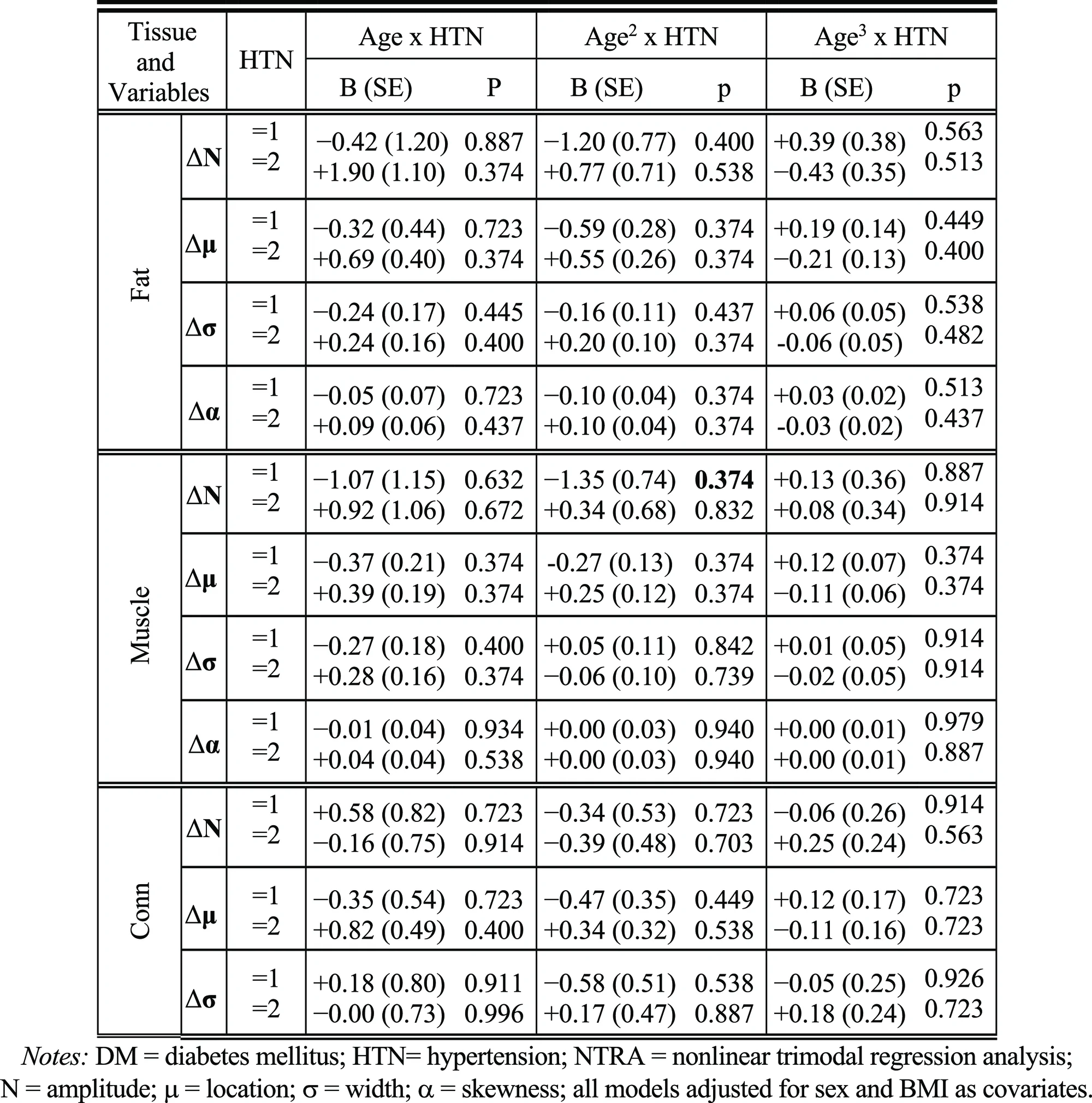
HTN status modifies linear associations of age and *Δ * NTRA parameters.

Tissue and Variables		HTN	Age x HTN		Age^2^ x HTN		Age^3^ x HTN	
			B (SE)	P	B (SE)	p	B (SE)	p
Fat	*Δ * N	*= 1=2*	−0.42 (1.20) + 1.90 (1.10)	0.887 0.374	−1.20 (0.77) + 0.77 (0.71)	0.400 0.538	+ 0.39 (0.38) −0.43 (0.35)	0.563 0.513
	$\Delta \mu $	*= 1=2*	−0.32 (0.44) + 0.69 (0.40)	0.723 0.374	−0.59 (0.28) + 0.55 (0.26)	0.374 0.374	+ 0.19 (0.14) −0.21 (0.13)	0.449 0.400
	$\Delta \sigma $	*= 1=2*	−0.24 (0.17) + 0.24 (0.16)	0.445 0.400	−0.16 (0.11) + 0.20 (0.10)	0.437 0.374	+ 0.06 (0.05) -0.06 (0.05)	0.538 0.482
	$\Delta \alpha $	*= 1=2*	−0.05 (0.07) + 0.09 (0.06)	0.723 0.437	−0.10 (0.04) + 0.10 (0.04)	0.374 0.374	+ 0.03 (0.02) -0.03 (0.02)	0.513 0.437
Muscle	*Δ * N	*= 1=2*	−1.07 (1.15) + 0.92 (1.06)	0.632 0.672	−1.35 (0.74) + 0.34 (0.68)	0.374 0.832	+ 0.13 (0.36) + 0.08 (0.34)	0.887 0.914
	$\Delta \mu $	*= 1=2*	−0.37 (0.21) + 0.39 (0.19)	0.374 0.374	-0.27 (0.13) + 0.25 (0.12)	0.374 0.374	+ 0.12 (0.07) −0.11 (0.06)	0.374 0.374
	$\Delta \sigma $	*= 1=2*	−0.27 (0.18) + 0.28 (0.16)	0.400 0.374	+ 0.05 (0.11) −0.06 (0.10)	0.842 0.739	+ 0.01 (0.05) −0.02 (0.05)	0.914 0.914
	$\Delta \alpha $	*= 1=2*	−0.01 (0.04) + 0.04 (0.04)	0.934 0.538	+ 0.00 (0.03) + 0.00 (0.03)	0.940 0.940	+ 0.00 (0.01) + 0.00 (0.01)	0.979 0.887
Conn	*Δ * N	*= 1=2*	+ 0.58 (0.82) −0.16 (0.75)	0.723 0.914	−0.34 (0.53) −0.39 (0.48)	0.723 0.703	−0.06 (0.26) + 0.25 (0.24)	0.914 0.563
	$\Delta \mu $	*= 1=2*	−0.35 (0.54) + 0.82 (0.49)	0.723 0.400	−0.47 (0.35) + 0.34 (0.32)	0.449 0.538	+ 0.12 (0.17) −0.11 (0.16)	0.723 0.723
	$\Delta \sigma $	*= 1=2*	+ 0.18 (0.80) −0.00 (0.73)	0.911 0.996	−0.58 (0.51) + 0.17 (0.47)	0.538 0.887	−0.05 (0.25) + 0.18 (0.24)	0.926 0.723

Notes: DM = diabetes mellitus; HTN = hypertension; NTRA = nonlinear trimodal regression analysis; N = amplitude; *μ =* location; *σ =* width; *α =* skewness; all models adjusted for sex and BMI as covariates.

**FIGURE 3.**
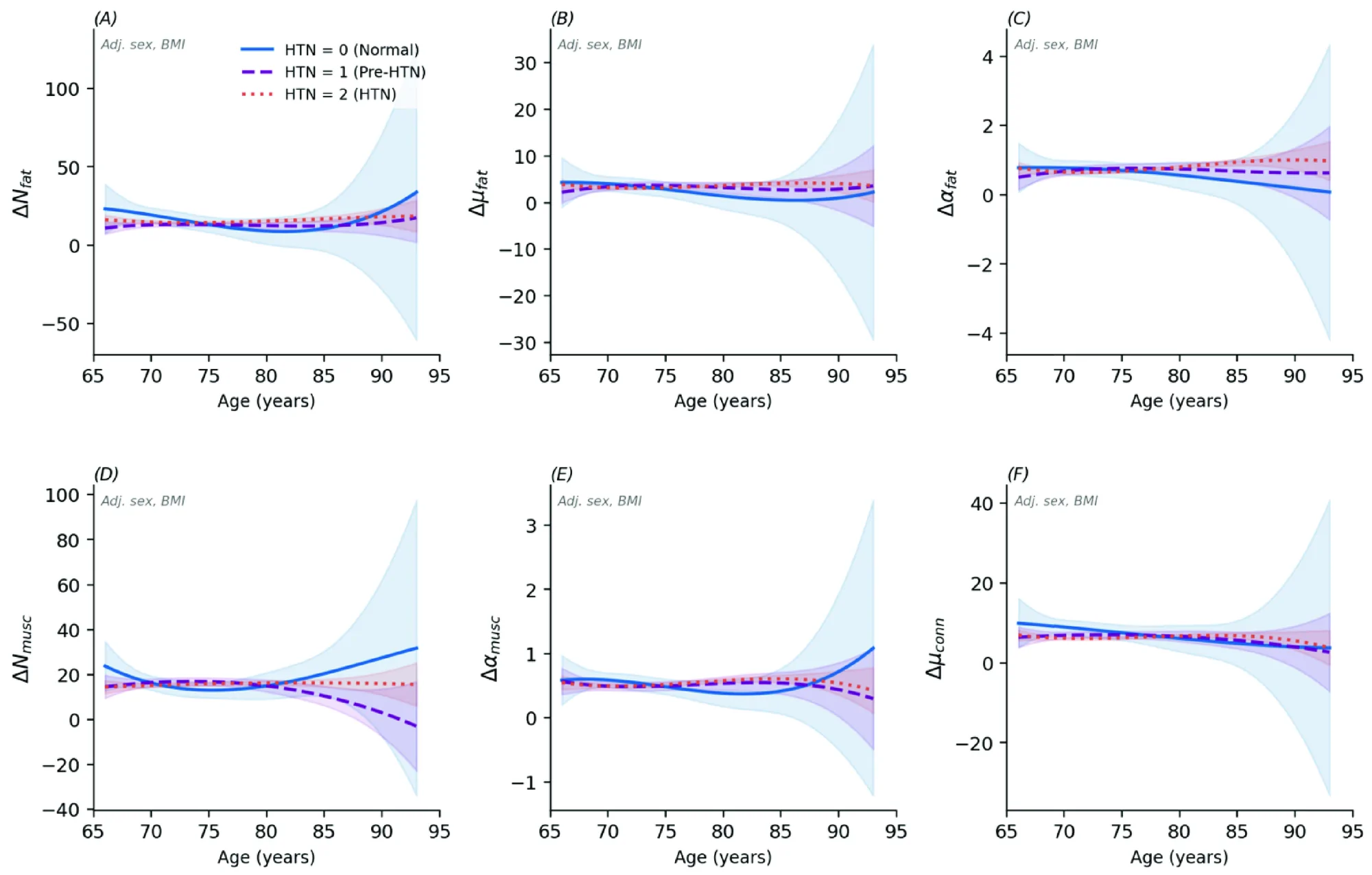
Associations between age and A), B), C), D), E), and F) stratified by HTN status. A third-order polynomial fit (solid lines) with 95% confidence intervals (shaded regions) is shown for each HTN group: HTN = 0 (normal, blue), HTN = 1 (pre-HTN, purple), and HTN = 2 (HTN, red).

**FIGURE 4.**
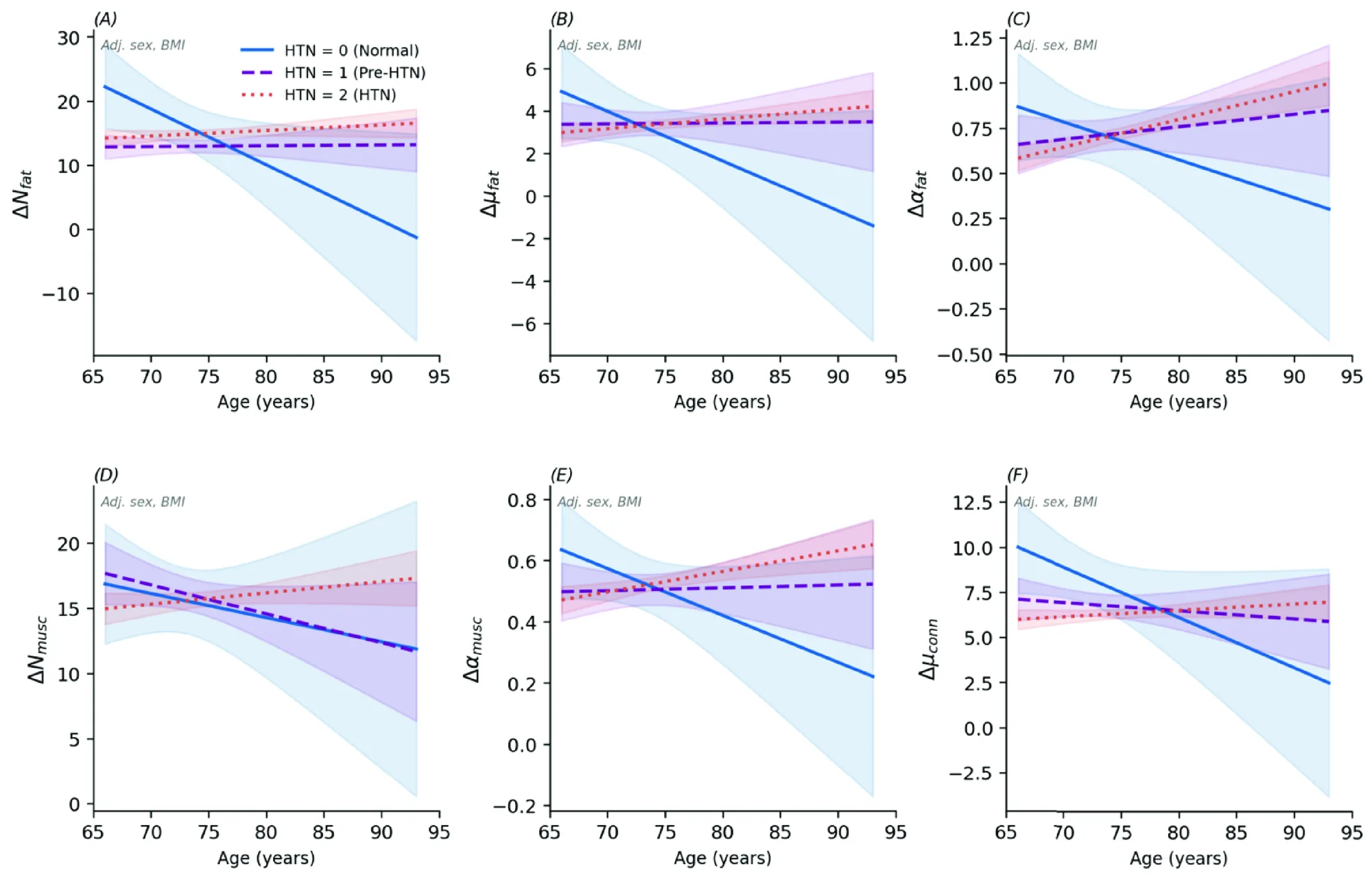
Associations between age and A), B), C), D), E), and F) stratified by HTN status. A linear polynomial fit (solid lines) with 95% confidence intervals (shaded regions) is shown for each HTN group: HTN = 0 (normal, blue), HTN = 1 (pre-HTN, purple), and HTN = 2 (HTN, red).

### C. NTRA Asymmetry Modifies Logistic Associations of Age-DM and Age-HTN

Table 4 shows logistic associations of NTRA asymmetry parameters with DM and HTN status, with all models adjusted for sex and BMI. Only one parameter was significantly associated with DM status: $\Delta \mu _{\text {fat}}$ (BH-adjusted p = 0.040). In contrast, two NTRA asymmetry parameters were significantly associated with HTN status: $\Delta \mu _{\text {conn}}$ and $\Delta \sigma _{\text {conn}}$ (both BH-adjusted p = 0.049). The logistic regression coefficient for $\Delta \mu _{\text {fat}}$ (B *= +0.025*) indicates a positive association between fat tissue location asymmetry and DM status after adjustment for age, sex, and BMI. The negative coefficients for connective tissue asymmetry in HTN models suggest that greater bilateral symmetry in connective tissue distribution may be associated with higher HTN burden, potentially reflecting systemic vascular stiffness that affects both limbs equivalently.

**TABLE 4:**
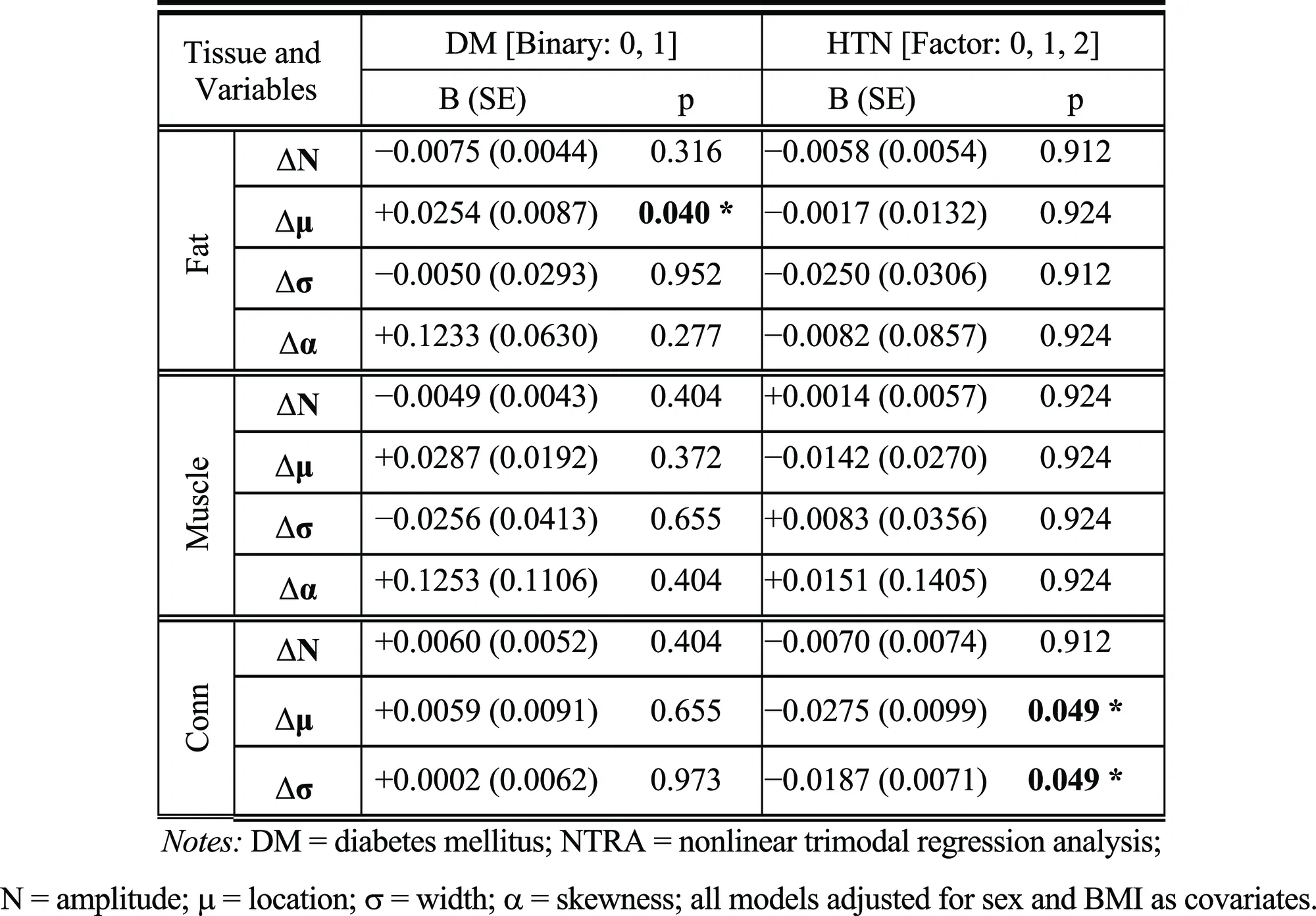
Logistic associations of *Δ * NTRA-DM and *Δ * NTRA-HTN.

Table 5 contains results from testing whether NTRA asymmetry modified the logistic association of age on DM status; no significant interaction effects were found. Table 6 contains results from testing whether NTRA asymmetry modified logistic associations of age on HTN status, with effects stratified by HTN group with reference to the control (HTN = 0).

**TABLE 5:**
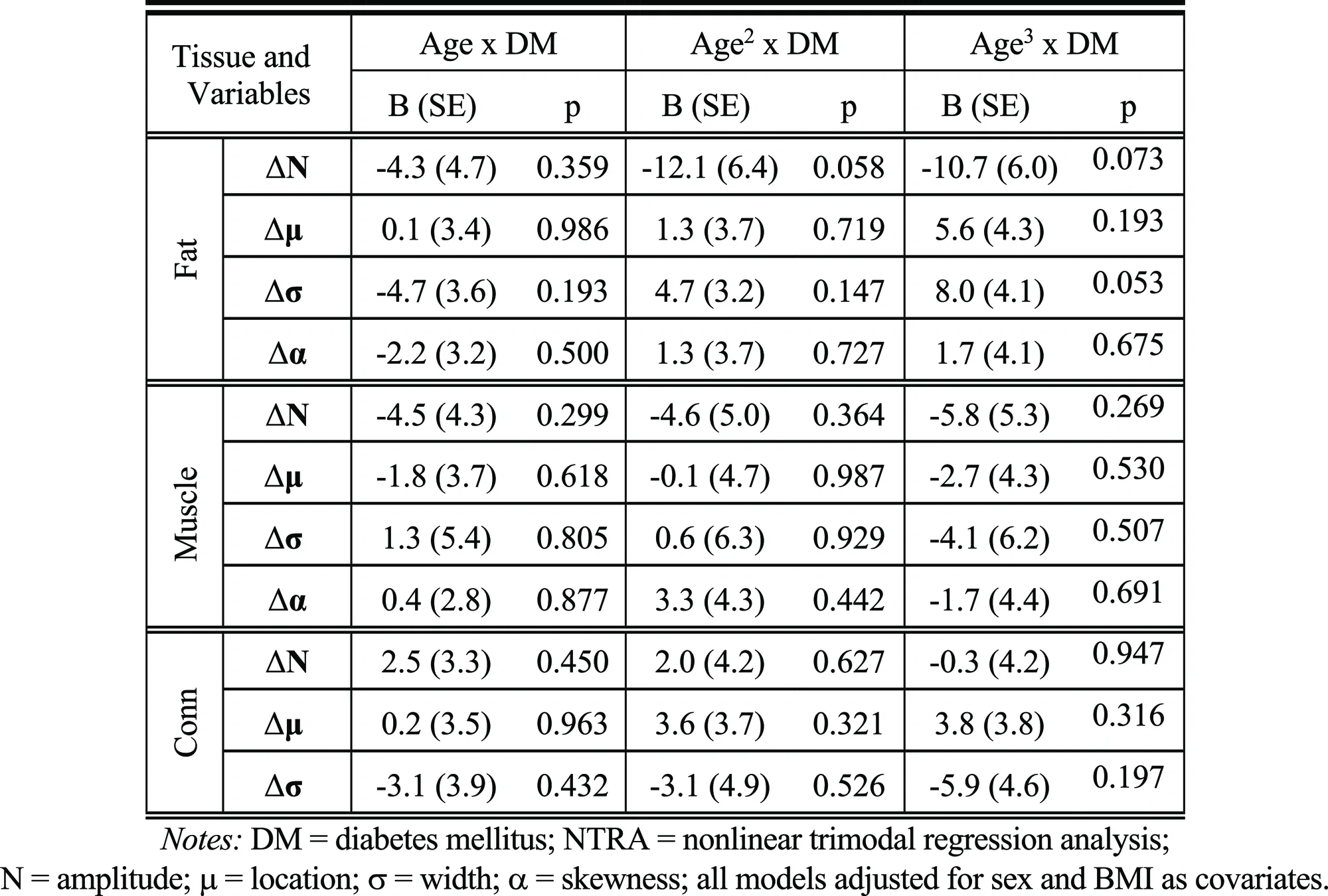
*Δ * NTRA parameters modify logistic associations of age and DM.

**TABLE 6:**
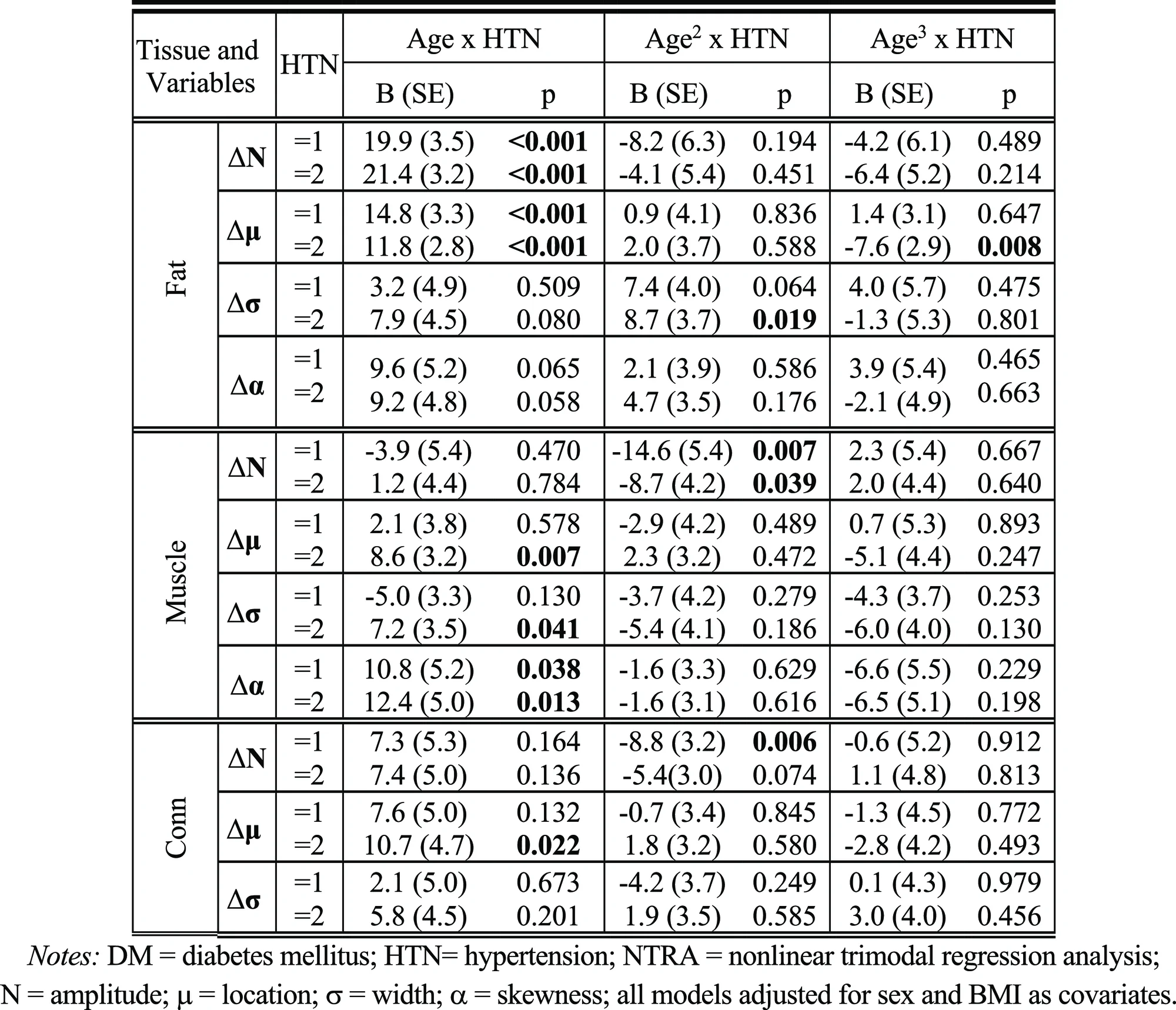
*Δ * NTRA parameters modify logistic associations of age and HTN.

As a sensitivity analysis, first-order (linear) polynomial fits were applied to the same age-NTRA associations stratified by disease status (Figs. 2B and 3, linear panels). The directional consistency between cubic and linear fits confirms that the reported stratification patterns are not artefacts of high-order polynomial fitting, but reflect genuine trends in the age-asymmetry relationship.

### D. Machine Learning Shows That NTRA Asymmetry Predicts DM and HTN Status

Table 7 presents classification metrics for all ML models. Among the five algorithms examined, RF and SVM demonstrated the highest and most comparable overall performance, while ADA-Boost showed markedly lower and inconsistent results. For DM classification, RF achieved an accuracy of 89.3% and an AUC-ROC of 95.8%; SVM achieved 88.3% accuracy and an AUC-ROC of 95.8%. For the three-class HTN classification, RF achieved 88.1% accuracy (weighted F1: 88.0%) and SVM achieved 87.8% (weighted F1: 88.0%). Metrics were obtained on SMOTE-balanced training datasets. Given the original class imbalance, a no-information classifier predicting the majority class would yield 89.6% and 78.9% accuracy for DM and HTN respectively; AUC-ROC and per-class F1-scores can therefore be considered the more informative evaluation criteria.

**TABLE 7:** ML models performances (%) with the use of SMOTE for binary classification of DM and HTN three-classes classification (weighted metrics). The most relevant results are printend in bold.

Comorbidity	Algorithm	Accuracy	Precision	Recall	F1	AUCROC
Diabetes	RF	*89.29 ± 1.09*	*86.86 ± 1.58*	*92.58 ± 1.60*	*89.63 ± 1.03*	*95.77 ± 0.68*
	GB	*79.21 ± 0.97*	*75.58 ± 1.41*	*86.32 ± 2.20*	*80.59 ± 0.91*	*86.90 ± 1.25*
	ADA-B	*65.38 ± 1.12*	*63.64 ± 0.94*	*71.77 ± 2.48*	*67.46 ± 1.36*	*70.74 ± 1.18*
	SVM	*88. 28 ± 0.98*	*94.16 ± 1.32*	*83.77 ± 2.33*	*88.66 ± 1.19*	*95.75 ± 0.57*
	KNN	*79.52 ± 1.25*	*71.34 ± 1.31*	*98.69 ± 0.35*	*82.81 ± 0.86*	*92.84 ± 1.25*
Hypertension	RF	*88.11 ± 1.93*	*88.07 ± 1.98*	*87.87 ± 1.94*	*87.97 ± 1.99*	-
	GB	*78.49 ± 1.72*	*78.40 ± 1.84*	*77.76 ± 1.79*	*78.08 ± 1.75*	-
	ADA-B	*52.49 ± 2.09*	*53.27 ± 2.11*	*52.05 ± 2.10*	*52.16 ± 2.15*	-
	SVM	*87.80 ± 1.23*	*88.88 ± 0.72*	*87.09 ± 1.21*	*87.98 ± 1.19*	-
	KNN	*80.06 ± 1.15*	*83.40 ± 1.22*	*73.13 ± 1.19*	*77.93 ± 1.41*	-

SVM = Support Vector Machine; KNN = K- Nearest Neighbor Notes: RF = Random Forest; GB = Gradient Boosting; ADA-B = ADA Boosting;

Per-class classification metrics for the RF model are reported in Table 8. For DM, the model achieved a precision of 0.691, recall of 0.882, and F1-score of 0.775 for the DM-positive class, indicating effective identification of the minority class under balanced conditions; the AUC-ROC of 0.813 confirms discriminative capacity beyond the class distribution. For HTN, per-class analysis reveals that the No-HTN class (original prevalence 5.2%) achieved the highest recall (0.892), while the pre-HTN class (original prevalence 16.0%) showed the lowest F1-score (0.610), reflecting the inherent difficulty of distinguishing the pre-hypertensive boundary. The established HTN class achieved intermediate recall (0.583). This pattern suggests that NTRA asymmetry is more sensitive to the presence versus absence of HTN than to differentiating its progression stages.

**TABLE 8:** Per-class classification mentrics.

Task / Class	Prev. (orig.)	Precision	Recall	F1-score
0 (No DM)	89.6%	0.837	0.606	0.703
1 (DM)	10.4%	0.691	0.882	0.775
Weighted avg	-	0.764	0.744	0.739
Overall	No-info = 89.6%	Acc = 0.744	AUC-ROC = 0.813	-
0 (No HTN)	5.2%	0.610	0.892	0.725
1 (Pre-HTN)	16.0%	0.713	0.533	0.610
2 (HTN)	78.8%	0.737	0.583	0.651
Weighted avg	-	0.687	0.669	0.662
Overall	No-info = 78.8%	Acc = 0.669	-	-

Notes: DM = Diabetes Mellitus HTN = Hypertension; Prev. (orig.): Prevalence of Classes in the original dataset

Feature importance analysis was conducted for all classification targets, excluding the ADA-Boost (ADA-B) algorithm due to its modest performance (Tables 9 and 10).

**TABLE 9:** Feature importance (%) for DM classification with RF and GB algorithm.

Feat	RF	GB
$\boldsymbol {\Delta } \textbf {N}$ Fat	10.02	13.17
$\boldsymbol {\Delta } \boldsymbol {\mu }$ Fat	8.73	7.46
$\boldsymbol {\Delta } \boldsymbol {\sigma }$ Fat	8.90	8.76
$\boldsymbol {\Delta } \boldsymbol {\alpha }$ Fat	8.93	7.39
$\Delta \textbf {N}$ Mus	9.74	10.19
$\boldsymbol {\Delta } \boldsymbol {\mu }$ Mus	8.17	6.87
$\boldsymbol {\Delta } \boldsymbol {\sigma }$ Mus	8.61	7.63
$\boldsymbol {\Delta } \boldsymbol {\alpha }$ Mus	8.80	8.75
$\Delta \text {N}$ Conn	8.58	8.98
$\boldsymbol {\Delta } \boldsymbol {\mu }$ Conn	9.55	9.53
$\boldsymbol {\Delta } \boldsymbol {\sigma }$ Conn	9.98	11.27

**TABLE 10:** Feature importance (%) for HTN classification with RF and GB algorithm.

Feat	RF	GB
$\boldsymbol {\Delta } \textbf {N}$ Fat	10.49	13.93
$\boldsymbol {\Delta } \boldsymbol {\mu }$ Fat	8.25	6.71
$\boldsymbol {\Delta } \boldsymbol {\sigma }$ Fat	9.05	8.53
$\boldsymbol {\Delta } \boldsymbol {\alpha }$ Fat	7.88	6.65
$\Delta \textbf {N}$ Mus	10.07	11.20
$\boldsymbol {\Delta } \boldsymbol {\mu }$ Mus	9.11	7.71
$\boldsymbol {\Delta } \boldsymbol {\sigma }$ Mus	9.37	9.00
$\boldsymbol {\Delta } \boldsymbol {\alpha }$ Mus	8.31	6.80
$\Delta \text {N}$ Conn	9.65	11.47
$\boldsymbol {\Delta } \boldsymbol {\mu }$ Conn	8.79	8.58
$\boldsymbol {\Delta } \boldsymbol {\sigma }$ Conn	9.03	9.41

In nearly all cases (with one exception), *Δ* **N_fat_** it was the most influential feature in both RF and GB models. *Δ* **N** also had substantial importance when derived from the other two soft tissues, contributing approximately one-third of the total importance for HTN classification and 36.4% DM classification with GB.

$\Delta \sigma $ ranked as the second most important geometric feature across all models, particularly in DM and HTN classifications from fat and connective tissue. For DM and HTN prediction, fat and muscle showed similar feature importance percentages. In table S-I of the Supplementary Material it is possible to access the selected best hyperparameters for the models which results are shown in table 7.

### E. Explainability

In Fig. 6, PDPs are presented for the RF algorithm in both classification tasks (i.e., DM and HTN prediction). Although, full overview and details on explainability analysis are reported in the Supplementary Material, here we make a focus on two representative asymmetry features chosen for both fat and muscle tissues. ${\Delta N}_{fat}$ is chosen because it represents the amplitude of the radiodensity distribution of fat tissues, thereby serving as an indicator of peaks in fat tissue amount. It also emerges as key parameter significantly associated with DM (as reported in the logistic regression analysis, see Table 4) and as the most important predictor in both DM and HTN classification (as reported in the feature importance analysis, see Fig. 4 and Fig. 5). The second feature and ${\Delta \sigma }_{musc}$, is chosen because it represents the standard deviation of the radiodensity distribution of muscle tissues, thereby serving as a measure of the heterogeneity of muscle tissue composition. As already reported in previous studies [31] and in Table 6 of the present work, it emerges as an indicator associated with HTN and with other risk factors, such as BMI and aging.

**FIGURE 5.**
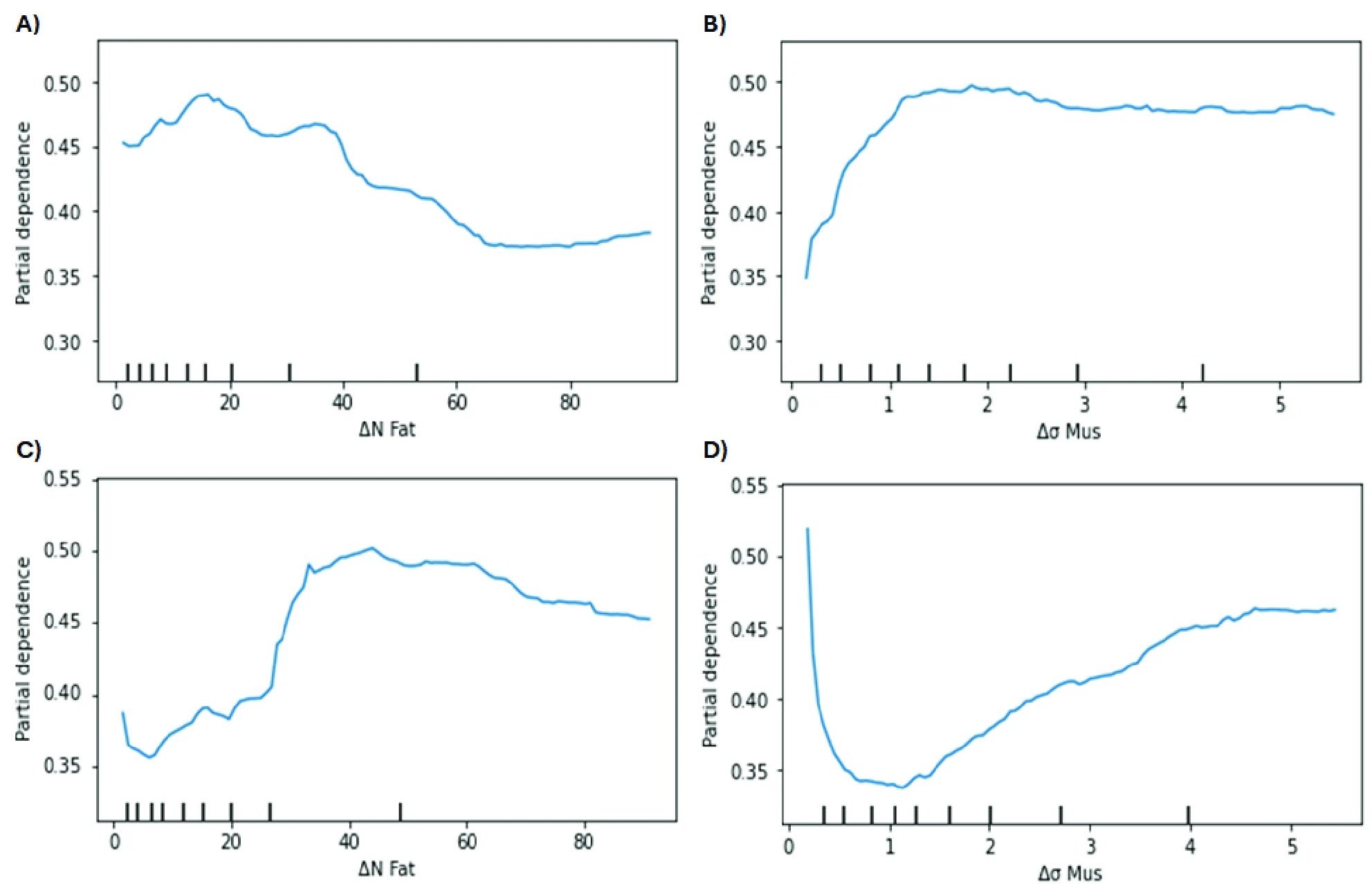
Partial dependence plots (PDPs) of the model with the highest accuracy (i.e., Random Forest as from Table 7) for (A-B) DM classification and (C-D) HTN classification (given the multiclass nature of the task, PDPs for HTN are shown in the case of class HTN = 2 prediction, i.e., hypertension class). Two representative features from fat (*Δ * Nfat: A-C) and muscle ($\Delta \sigma $ mus: B-D) tissues are reported. Complete overview of PDPs for all features, algorithms and classification tasks are available in the supplementary material.

While in Fig. 6A and Fig. 6B the probabilities of having DM (class DM = 1) are displayed as a function of two selected features for both fat (${\Delta N}_{fat}$, in Fig. 6A) and muscle (${\Delta \sigma }_{musc}$, in Fig. 6B) tissues, in Fig. 6C and 6D, PDPs are presented for HTN = 2 class (i.e., class of subjects with HTN), thus representing the probability of predicting HTN as a function of two features for both fat (${\Delta N}_{fat}$, in Fig. 6C) and muscle (${\Delta \sigma }_{musc}$, in Fig. 6D) tissues. It can be observed that, according to the implemented RF model, in the case of both DM and HTN prediction, high asymmetry in the heterogeneity of muscle (${\Delta \sigma }_{musc}$) tissue composition contributes to increasing probability of belonging to the diseased group. Conversely, and opposite behavior in the asymmetry of peak fat tissue (${\Delta N}_{fat}$) is observed between the DM and HTN case. Indeed, an inverse proportion is observed between ${\Delta N}_{fat}$ and probability of DM prediction (increased asymmetry decreases DM prediction probability), while a direct proportion is observed between ${\Delta N}_{fat}$ and probability of HTN prediction (increased asymmetry increases HTN prediction probability). Note that the feature axes in PDP and SHAP plots are expressed in the native units of the NTRA asymmetry parameters, for which established clinical thresholds do not currently exist: these visualizations serve as model-interpretation tools only.

These results, taken together with the additional analyses and plots reported in the Supplementary Material (see Figures from S-1 to S-6), reveal also the role of ${\Delta \mu }_{fat}$ as a significant asymmetry indicator in both DM and HTN prediction. They confirm that the adopted classification algorithms were able to capture the significant relationships between the risk of developing DM or HTN and the unbalances in fat and muscle tissue composition and heterogeneity, particularly reflected by the $\Delta \sigma _{\text {musc}}$ indicator. Indeed, as far as DM prediction, it appears that high asymmetry in both fat and muscle tissues composition contribute to increasing DM probability (Figures S-1 and S-5). Similarly, for the case of HTN prediction, the probability of being in the HTN = 0 class (i.e., no HTN) shows a remarkable decrease with asymmetry indicators of fat and muscle (Figures S-2 and S-6 A). Accordingly, probabilities of being in classes HTN = 1 and HTN = 2 increase at increasing asymmetry (Figures S-3, S-4, S-6 B).

## IV. Discussion

### A. Summary of the Results

Using cross-sectional data from the AGES-Reykjavík study, this work investigated the role of soft tissue radiodensity asymmetry using the NTRA features in classifying DM and HTN status among older community-dwelling adults, supporting their potential role as opportunistic imaging biomarkers for metabolic and vascular risk stratification in future translational studies. A key distinction from prior work by our group [30], [31] is that the current study investigates bilateral asymmetry of radiodensity distributions, rather than absolute single-leg NTRA values, as potential biomarkers for HTN and DM. While [30] demonstrated that single-leg parameters are predictive of DM and HTN, the directionality and magnitude of inter-limb differences, which may reflect localized sarcopenic processes or asymmetric vascular dysfunction, had not previously been explored in the context of metabolic disease. Our previous work with NTRA asymmetry features was limited to only functional mobility and gait characterization of the AGES elderly population [31]. Nonetheless, given that both studies draw on the same cohort and feature set, the risk of cumulative overfitting must be acknowledged.

Regression analyses revealed that DM and HTN status significantly modify the relationship between age and NTRA asymmetry parameters, with distinct effects observed for either condition. For DM, muscle tissue asymmetry (${\Delta \mu }_{musc}$) showed a significant interaction with age, suggesting that muscle-specific changes may reflect disease-related alterations in tissue composition. In contrast, for HTN, directional trends were observed across multiple NTRA parameters spanning all three tissue types; however, no interaction term survived adjustment for sex, BMI, and Benjamini-Hochberg correction, suggesting these patterns should be interpreted as exploratory rather than confirmatory: broader disruptions in tissue composition due to HTN progression highlighted in elderly population are therefore not seen from our results [40].

Logistic regression further identified fat tissue asymmetry ($\Delta \mu _{\text {fat}}$) as significantly associated with DM, and connective tissue asymmetry ($\Delta \mu _{\text {conn}}$ and $\Delta \sigma _{\text {conn}}$) as key predictors of HTN, after covariate adjustment for sex and BMI.

ML classification models further demonstrated that amplitude (*Δ * N) and width ($\Delta \sigma $) asymmetry parameters effectively differentiate between DM and HTN participants. Among the five algorithms examined, RF and SVM showed comparable top performance. Feature importance analysis highlighted fat tissue amplitude asymmetry (*Δ * N${}_{\text {fat}}$) as the most influential predictor for both DM and HTN classification.

### B. Moderation and Logistic Association of Age, NTRA Asymmetry and DM/HTN Status

Results from examining moderation and logistic associations of age, NRTA asymmetry, and DM/HTN status altogether highlight the complex interplay between these variables, with asymmetry parameters appearing to amplify or attenuate the age-HTN relationship depending on the tissue type. Specific tissue types, particularly fat and connective tissues, may play unique roles in disease development and progression. Approximately 40% of total human body weight is skeletal muscle [41], which is influenced by various factors such as nutritional status, hormonal balance, physical activity or exercise, and the presence of disease [42], [43], [44]. Skeletal muscle plays a crucial role not only in maintaining posture and enabling movement but also in regulating glucose and protein metabolism [43], [45]. Given that skeletal muscle is the primary site for glucose disposal, a decline in muscle mass can lead to impaired glucose metabolism, raising the risk of DM in older adults [46]. Pathological variations in muscle attenuation, reflecting excessive intramuscular fat accumulation, are frequently observed in individuals with obesity and type II diabetes [47].

Age-related changes in skeletal muscle, such as muscle atrophy or increased fat mass, are key factors for the elevated risk of DM in older adults [10], [44], [48]. Studies have further shown that DM is associated with an increased likelihood of mobility disability compared to non-diabetics [49]. Those with DM face a two to threefold higher risk of physical disability [50], [51]. Maintaining muscle quality and strength is crucial for metabolic health, as declines in muscle quality are significant risk factors for older adults [52]. Older adults with DM require tailored care with glycemic targets and management according to their functional status [53], [54] as they may experience several leg-related conditions, including peripheral neuropathy which can potentially affect balance and increase the risk of falls [55].

The current study found significant differences in the *Δ N* and $\Delta \sigma $ asymmetry parameters from muscle tissues for DM classification, which may indicate that uneven muscle mass loss that can impair glucose disposal capacity, as also supported by the results of the explainability analysis that unveil the direct proportional associations between $\Delta \sigma $ asymmetry from muscle tissues and DM prediction. The explainability results complement observations from a previous study [31], which suggested that values of mean asymmetry of fat and muscle tissues, as measured from radiodensity distributions in mid-thigh CT cross-sections, are higher in subjects with high BMI, which is a well-recognized risk-factor in both type-2 DM and HTN. BMI was included as a covariate in the regression analyses. Its influence on soft tissue radiodensity distributions, and consequently on asymmetry indices, is acknowledged; however, the fact that significant associations persist after BMI adjustment suggests that asymmetry captures independent variation beyond that explained by overall body composition. Physiologically, the observed asymmetry in muscle tissue may reflect localized sarcopenic processes and heterogeneous myosteatosis, which are common in individuals with metabolic disorders. In diabetes, insulin resistance reduces glucose uptake primarily in skeletal muscle, the largest insulin-sensitive tissue, leading to selective atrophy and infiltration of fat in certain muscle regions [30], [56]. Furthermore, these findings underscore the potential of leg muscle tissue asymmetry as a predictive biomarker for DM risk, but further investigation into mediating mechanisms is required for deeper insight into the metabolic imbalances associated with aging.

### C. ML Algorithm Comparison and Classification of DM

Findings from ML models showed that the RF algorithm exhibited superior performance in classifying both DM and HTN status. This aligns with previous studies that have shown excellent evaluation metrics in RF’s classification of either DM or HTN [30]. Overall, the high classification scores from ML models indicate that these algorithms are highly effective at distinguishing between subjects with or without DM, emphasizing the effectiveness of incorporating NTRA asymmetry features for predicting metabolic disorders in aging populations.

Among the five ML algorithms evaluated, RF demonstrated the highest overall performance for DM classification (accuracy: 89.3%, AUC-ROC: 95.8%), with SVM achieving comparable results (accuracy: 88.3%, AUC-ROC: 95.8%). These metrics were obtained on a SMOTE-balanced training set and should be interpreted as upper-bound estimates, reflecting feature-space separability under balanced conditions. To contextualize these results more precisely, per-class analysis (Supplementary Table S-I) reveals that for the DM-positive class, RF achieved a precision of 0.691, recall of 0.882, and F1-score of 0.775 on the balanced test set, indicating that the model learns to identify the minority DM class effectively under balanced conditions. The AUC-ROC of 0.813 further confirms that the asymmetry features provide discriminative value beyond chance, even when evaluated without balancing assumptions. These findings are consistent with prior studies demonstrating strong RF performance in metabolic disorder classification using NTRA features [30].

### D. ML Algorithm Comparison and Classification of HTN

Unlike DM, NTRA asymmetry values showed a more limited capability to differentiate among the three HTN classes. RF achieved an accuracy of 88.1% (weighted F1: 88.0%) with SMOTE. Per-class analysis (Table 8) reveals important differences across HTN groups: the No-HTN class (original prevalence 5.2%) achieved the highest recall (0.892), while the Pre-HTN class (original prevalence 16.0%) showed the lowest F1-score (0.610), reflecting the inherently ambiguous boundary between pre-HTN and established HTN. The established HTN class (original prevalence 78.8%) achieved intermediate recall (0.583). This pattern suggests that NTRA asymmetry is a more sensitive marker for identifying the presence versus absence of HTN than for differentiating its progression stages, consistent with the observation that the initial onset of HTN may produce more distinct tissue compositional changes than the transition from pre-HTN to established HTN [30].

It is important to note that the high overall accuracy primarily reflects the RF model’s proficiency with the majority HTN class, and that the weighted F1 metric provides a more balanced summary of multi-class performance. The association between skeletal muscle decline and HTN is well established [57], [58], involving interactions between muscle composition, vascular function, and overall body composition [13], [57], [59]. While NTRA parameters are sensitive to these compositional changes, bilateral asymmetry appears to be a less discriminating marker for monitoring HTN progression across classes, warranting further investigation.

A notable discrepancy exists between the modest number of statistically significant asymmetry parameters identified through classical regression and the classification performance of ML models. This pattern is consistent with ensemble methods’ ability to capture weak, multivariate, non-linear interactions that do not survive BH correction in individual analyses. It also partly reflects the effect of SMOTE balancing on the feature space. These observations underscore the complementary nature of statistical and ML approaches in biomarker discovery.

### E. NTRA Amplitude and Fat Tissue Parameters as Key Contributors

Fat tissue asymmetry emerged as a key contributor across multiple analyses: $\Delta \mu _{\text {fat}}$ was the only fat-derived parameter to reach significance in logistic regression, specifically as a predictor of DM status (BH-adjusted p = 0.040), while *Δ * N_fat_ consistently ranked as the most influential feature in ML classification for both DM and HTN (Tables 9 and 10). Directional trends involving fat asymmetry parameters were also observed in age-HTN moderation models (Fig. 3 and 4), though none survived BH correction after covariate adjustment. These results align with prior evidence linking dysfunctional adipose tissue to metabolic disturbances, including insulin resistance and cardiovascular diseases [60], [61].

These findings underscore the particular importance of fat tissue asymmetry in predicting metabolic conditions. In general, adipose tissue stores fat molecules in the normal maintenance of metabolic balance [62]; however, when adipose tissue becomes dysfunctional, it can lead to a pro-inflammatory state, marked by increased insulin resistance type 2 DM, and cardiovascular diseases [60], [61], [62], [63]. Visceral adiposity has been further identified as a major independent risk factor for metabolic syndrome [60], [62]. Nevertheless, the biological mechanisms that underly the role of adipose tissue in aging health outcomes are still being investigated. Future studies should explore longitudinal changes in NTRA asymmetry to determine whether these parameters predict disease onset or progression over time.

### F. Clinical Impact

The proposed NTRA asymmetry framework is envisioned as a lightweight computational module for opportunistic risk stratification, designed to operate on CT images already acquired for other clinical purposes. In a prospective deployment scenario, the pipeline would automatically extract bilateral mid-thigh radiodensity distributions from DICOM-format CT data, compute the 11 asymmetry indices, and generate a risk flag for metabolic and vascular conditions to support clinical decision-making, without requiring additional imaging, radiation exposure, or patient burden.

From a clinical workflow perspective, the approach requires only a single low-dose CT acquisition of the lower extremities, with minimal additional time or computational resources. The ML algorithms employed are lightweight and executable on standard workstations without high-end infrastructure, and the pipeline is compatible with existing radiological segmentation practices. Individuals flagged by the system could be directed toward preventive care pathways, including metabolic screening, lifestyle interventions, or targeted therapies, prior to the emergence of traditional clinical symptoms or laboratory abnormalities, enabling a more proactive and personalized approach to managing age-associated chronic disease. An additional clinical applicability is the longitudinal use of the NTRA framework for patient monitoring. By exploiting repeated CT scans from routine clinical follow-up, the pipeline could track temporal changes in radiodensity asymmetry and detect early deviations from individual baseline trajectories, enabling the identification of subclinical deterioration before clinical manifestation.

From a regulatory standpoint, this tool would likely be classified as a Class IIa Software as a Medical Device (SaMD) under EU-MDR 2017/745, given its role as a decision-support aid complementing, rather than replacing, established diagnostic standards. The pipeline operates exclusively on anonymized, imaging-derived features, ensuring compliance with GDPR without requiring sensitive patient metadata.

The performed explainable ML methods (PDP and SHAP) transform model outputs from opaque predictions into quantifiable, feature-level contributions. This process enables clinicians to interpret and critically assess the underlying decision-making process. This interpretability is essential to foster trust, support clinical validation, and facilitate the integration of AI-driven recommendations into routine practice.

Furthermore, the clinical applicability of the proposed approach has already been demonstrated in a real-world context by our research group. In a recent case study, 2D and 3D CT-derived radiodensity parameters were used to monitor the effects of long-term, home-based physical exercise in an older adult, showing preserved muscle quality and reduced fat infiltration highlighting the potential application of these radiodensity biomarkers in personalized strategies to mitigate sarcopenia and related metabolic complications [64]. Moreover, the translational potential of the method has been confirmed in the cardiovascular domain focusing on using CT imaging to perform the concept of “virtual cardiac histology” characterizing left ventricular tissue by analyzing radiodensitometric distributions (density variations) to distinguish between healthy and diseased cardiac muscle [65]. Similarly, radiodensity assessment via CT has shown clinical value in diverse populations, with lower values consistently associated with worse outcomes such as increased postoperative complications, reduced cancer survival, and impaired functional status in the elderly [66]. Counteracting these mechanisms with easy-to-perform and safe full-body in-bed gymnastics exercises for rejuvenation in older people can significantly contribute to the prevention of related risks [72], [73]. These applications collectively position NTRA-based asymmetry analysis as a versatile, scalable tool for embedding musculoskeletal biomarkers into routine clinical workflows.

### G. Limitations and Future Directions

This study offers valuable insights into the use of soft tissue radiodensity asymmetry as predictive biomarkers for DM and HTN. However, some limitations must be acknowledged. This analysis uses data from the AGES-Reykjavik cohort, a well-established resource for aging studies [67], [68]. However, the cohort predominantly includes older adults of Icelandic descent living in a relatively homogeneous socio-environmental context. This demographic and environmental uniformity, encompassing shared ethnicity, lifestyle habits, comorbidity profiles, and healthcare access, may limit the generalizability of the findings to more diverse populations. Additionally, certain aging trajectories observed in this cohort may reflect population-specific genetic or cultural factors that do not extend to other settings. Future studies involving more diverse populations across different ethnic, geographic, and healthcare contexts will be essential to validate these findings and assess their broader applicability.

The cross-sectional design precludes causal inference and limits our ability to determine whether soft tissue asymmetry changes precede the onset of DM or HTN. Consequently, the observed associations between asymmetry parameters and disease status reflect contemporaneous relationships, and it remains unknown whether asymmetry is a cause, consequence, or concomitant of these conditions. Longitudinal investigations are needed to evaluate the predictive value of these biomarkers over time and their utility in monitoring disease progression. Future work should also integrate NTRA-derived parameters with other routinely collected clinical data to develop more comprehensive models for multimorbidity management [69], [70]. Although sex and BMI were included as covariates, additional potential confounders, including physical activity level, medication use, and comorbidity burden, were not available for all AGES participants. These represent residual confounding factors that should be addressed in future studies. Further improvements could also include inflammatory markers and hormonal variables to better characterize the biological mechanisms underlying the observed asymmetry patterns.

Importantly, the present work proposes an interpretable analytical framework for investigating the relationship between chronic disease status and NTRA-derived biomarkers, whose relevance has been supported by previous studies. Although the identified associations are biologically plausible and partially consistent with existing evidence, the predictive performance of the ML models remains strongly influenced by the marked class imbalance of the dataset and the consequent reliance on balancing approaches such as SMOTE. While cross-validation was used to mitigate overfitting, synthetic resampling may still affect model behaviour and performance estimates. Therefore, the reported metrics should be interpreted cautiously, as they may represent an upper-bound estimate of the model’s generalization ability and may not fully reflect real-world predictive performance. The findings should thus be considered primarily hypothesis-generating and exploratory, requiring independent validation before any potential clinical implementation can be considered.

The observed differences compared to traditional statistical analyses may reflect the ability of ML models to capture subtle, non-linear patterns that may not emerge through conventional statistical approaches [71]. However, whether these patterns represent robust and generalizable disease-related signals remains to be determined. Future work should therefore prioritize external validation on independent datasets, including populations with different demographic and clinical characteristics, as well as the evaluation of alternative classification frameworks that are less dependent on synthetic resampling procedures. Such efforts will be essential to improve model robustness, reduce the risk of overfitting, and establish the clinical relevance of the identified biomarkers.

Although the current study was not deployed in a real-world clinical setting, we recognize that any future translation into practice would require continuous monitoring and periodic revalidation using newly acquired data. Such procedures would help assess model stability over time, detect potential data drift, and ensure sustained reliability, fairness, and clinical utility across evolving patient populations.

Tree-based feature importance measures are known to exhibit bias toward high-variance and correlated features. Furthermore, the stability of importance rankings across cross-validation folds was not formally assessed in the present study. These limitations should be addressed in future analyses through approaches such as permutation-based importance, stability selection procedures, or other explainability techniques designed to improve the robustness and reproducibility of feature interpretation.

Finally, while associations between asymmetry features and disease states were identified, the underlying biological mechanisms remain incompletely understood. The observed asymmetry patterns may reflect complex interactions among metabolic, vascular, inflammatory, and age-related processes that cannot be fully disentangled within the current study design. Future investigations should focus on biological validation of these findings, including mechanistic studies and prospective clinical investigations incorporating additional confounding variables such as diet, physical activity, medication use, inflammatory status, hormonal variation, and metabolic disorders. Such efforts will be necessary to clarify the physiological significance of NTRA-derived biomarkers and their potential role in precision medicine applications.

## V. Conclusion

This study provides evidence that soft tissue radiodensity asymmetry parameters, derived from mid-thigh CT imaging using the NTRA method, are associated with DM and HTN status in older adults, supporting their potential role as opportunistic imaging biomarkers for metabolic and vascular risk stratification. Regression analyses confirmed that DM and HTN status significantly modify age-asymmetry relationships, particularly for muscle tissue in DM and across multiple tissue types in HTN, while logistic regression identified fat and connective tissue asymmetry as independent predictors of DM and HTN status respectively, robust to adjustment for sex and BMI. ML classification models reaffirmed the discriminative capacity of NTRA asymmetry features, particularly amplitude (*Δ * N) and fat tissue parameters, under balanced class conditions.

Taken together, these findings highlight the potential of tissue-specific radiodensity asymmetry as a novel, non-invasive, and computationally efficient biomarker that could be embedded into routine CT workflows for early risk identification in aging populations. Future work should focus on longitudinal validation, external cohort replication, and prospective clinical integration studies to further assess real-world generalizability and clinical utility.

## VI. Conflicts of Interest

All the authors declare no conflict of interest.

## VII. Author Contributions

Conceptualization, Methods: Marco Recenti, Alfonso M. Ponsiglione, Carlo Ricciardi, and Kyle J. Edmund; Software, Validation: Marco Recenti, Alfonso M. Ponsiglione, Carlo Ricciardi, and Michela Russo; Formal Analysis: Marco Recenti, Alfonso M. Ponsiglione, Carlo Ricciardi, Kyle J. Edmund, Magnus K. Gislason, Ugo Carraro, Milan Chang, and Paolo Gargiulo; Visualization: Marco Recenti, Alfonso M. Ponsiglione, Carlo Ricciardi, and Kyle J. Edmund; Writing, Original Draft: Marco Recenti, Alfonso M. Ponsiglione, Carlo Ricciardi, Michela Russo, and Kyle J. Edmund; Writing, Review, and Editing: Francesco Amato, Magnus K. Gislason, Ugo Carraro, Milan Chang, and Paolo Gargiulo; and Supervision, Project Administration: Paolo Gargiulo.

## VIII. Ethical Considerations

All NTRA participants provided written informed consent, and the study received ethical approval from the Icelandic National Bioethics Committee and the Reykjavík University Code of Ethics (Paragraph III of Article 2 of the Higher Education Institution Act No. 63/2006).

## Data Availability Statement

The datasets presented in this article are not readily available because the Reykjavik-AGES I-II dataset cannot be made publicly available, since the informed consent signed by the participants prohibits data sharing on an individual level, as outlined by the study approval by the Icelandic National Bioethics Committee (RU Code of Ethics, Paragraph III-Article 2 Higher Education Institution Act 63/2006). Requests to access the datasets should be directed to Hjartavernd: afgreidsla@hjarta.is afgreidsla@hjarta.is

## Supplementary Materials

Supplementary Materials
