# Supplementary material for: Soft Tissue Radiodensity Asymmetry as Clinical Indicator for Diabetes and Hypertension in Aging
Source: IEEE J Transl Eng Health Med. 2026 Jul 31;14:352–68. doi: 10.1109/JTEHM.2026.3718689 (PMC13750829; doi:10.1109/JTEHM.2026.3718689)
Supplement: Supplementary file 1 [file supp1-3718689.docx]

SUPPLEMENTARY MATERIAL

**Soft Tissue Radiodensity Asymmetry as**

**Clinical Indicator for Diabetes and**

**Hypertension in Aging**

**Marco Recenti, Alfonso M. Ponsiglione, Carlo Ricciardi, Michela Russo, Kyle J. Edmunds, Francesco Amato, Magnus K. Gislason, Ugo Carraro, Milan Chang, and Paolo Gargiulo**

Hyperparameter tuning

Hyperparameter tuning and optimization was carried out using Grid Search algorithm and the results in terms of optimal hyperparameters selected for the best-performing models, i.e., Random Forest (RF) and Gradient Boosting (GB), used for both the binary (type-2 Diabetes Mellitus, DM, prediction) and three-classes (Hypertension, HTN, prediction) classification tasks are reported in the following Table S-1.

**Table S-I**

ML models most performing hyperparameters (%) with the use of SMOTE for binary classification of DM and HTN three-classes classification (weighted metrics), evaluation metrics relative to these model are in table VII in the main text.

| **Comorbidity** | RF | GB | ADA-B | | SVM | KNN |
| --- | --- | --- | --- | --- | --- | --- |
| **Diabetes** | n_estimators: 100  max_depth: None  min_sample_leaf: 1  min_sample_split: 2 | n_estimators: 100  learning_rate: 0.1  max_depth: 10  min_sample_leaf: 1  min_sample_split: 2 | | n_estimators: 100  learning_rate: 0.1  base_estimator: Decision Tree | C: 1  Kernel: rbf  Gamma: scale | n_neighbors: 3  weight: uniform  algorithm: auto |
| **Hypertension** | n_estimators: 100  max_depth: 10  min_sample_leaf: 1  min_sample_split: 2 | n_estimators: 100  learning_rate: 0.1  max_depth: 5  min_sample_leaf: 1  min_sample_split: 2 | | n_estimators: 100  learning_rate: 0.1  base_estimator: Decision Tree | C: 1  Kernel: rbf  Gamma: scale | n_neighbors: 5  weight: uniform  algorithm: auto |

Notes: RF= Random Forest; GB= Gradient Boosting; ADA-B= ADA Boosting;

SVM= Support Vector Machine; KNN= K- Nearest Neighbor

No-SMOTE Results

To provide a fully comprehensive overview of the results, we report in the Table S-II below the models performances for DM and HTN classification without the use of SMOTE.

**Table S-II**

ML models performances (%) without the use of SMOTE for binary classification of DM and HTN three-classes classification (weighted metrics).

| **Comorbidity** | **Algorithm** | Accuracy | Precision | Recall | F1 | AUCROC |
| --- | --- | --- | --- | --- | --- | --- |
| **Diabetes** | **RF** | 86.18 ± 0.23 | 20.00 ± 40.00 | 0.23 ± 0.48 | 0.47 ± 0.94 | 51.01 ± 1.04 |
|  | **GB** | 85.59 ± 0.23 | 18.00 ± 15.18 | 1.19 ± 1.06 | 2.22 ± 1.98 | 52.64 ± 1.67 |
|  | **ADA-B** | 85.03 ± 0.69 | 12.22 ± 11.19 | 0.71 ± 0.58 | 1.34 ± 1.09 | 50.92 ± 2.86 |
|  | **SVM** | 86.12 ± 0.06 | 0.00 ± 0.00 | 0.00 ± 0.00 | 0.00 ± 0.00 | 53.98 ± 2.52 |
|  | **KNN** | 82.31 ± 0.06 | 13.61 ± 1.31 | 5.94 ± 2.87 | 7.51 ± 3.86 | 52.84 ± 3.81 |
| **Hypertension** | **RF** | 78.20 ± 0.26 | 61.34 ± 0.25 | 78.20 ± 0.25 | 68.76 ± 0.23 | - |
|  | **GB** | 78.07 ± 0.36 | 61.32 ± 0.24 | 78.07 ± 0.36 | 68.69 ± 0.25 | - |
|  | **ADA-B** | 78.27 ± 0.23 | 68.79 ± 0.24 | 78.27 ± 0.23 | 68.79 ± 0.24 | - |
|  | **SVM** | 78.34 ± 0.16 | 61.37 ± 0.25 | 78.34 ± 0.16 | 68.82 ± 0.22 | - |
|  | **KNN** | 72.57 ± 1.52 | 65.24 ± 1.70 | 72.57 ± 1.52 | 68.26 ± 1.31 | - |

*Notes:* RF= Random Forest; GB= Gradient Boosting; ADA-B= ADA Boosting;

SVM= Support Vector Machine; KNN= K- Nearest Neighbor

Explainbility analysis

For the best-performing models, explainability analysis [1] has been carried out by means of Partial Dependence Plots (PDPs), a model agnostic global method to explain the effect of one or more features on the overall predictive model outcome by considering marginal effects [2], and SHapley Additive exPlanations (SHAP), a model agnostic local method for correlating the probability of a predicted class to the value of a particular feature by calculating the average of the marginal contribution of a specific feature to the class prediction across all possible permutations of the features [3].

In particular, PDPs were derived to explain the input-output relationships of the classification algorithm for each of the following 11 Nonlinear Trimodal Regression Analysis (NTRA) asymmetry indicators (ΔNTRA, see equation 2 of the main manuscript for the definition of ΔNTRA):

- fat tissue asymmetry (ΔNfat, ∆μfat, Δσfat, ∆αfat)
- muscle tissue asymmetry (∆Nmus, ∆μmus, Δσmus, ∆αmus)
- connective tissue asymmetry (ΔNconn, Δμconn, Δσconn)

In the following figures (from S-1 to S-4), PDPs are presented for both RF and GB and for both classification tasks: (i) binary classification for DM prediction; (ii) three-classes classification for HTN prediction. In the former task (DM prediction), PDPs for display the probability of having DM (class DM = 1) as a function of each input feature value. In the latter task (HTN prediction), instead, PDPs are presented separately for each of the three classes; in particular, PDPs are displayed for HTN = 0 (Figure S-2), i.e., for subjects without hypertension, representing the probability of predicting HTN = 0 as a function of each input feature value; then PDPs are presented for HTN = 1 (Figure S-3), i.e., for subjects with pre-hypertension, defined as systolic blood pressure of 120–139 mmHg or diastolic blood pressure of 80–89 mmHg, representing the probability of predicting HTN = 1 as a function of each input feature value; finally, PDPs are also displayed for HTN = 2 (Figure S-4), i.e., for subjects with hypertension, representing the probability of predicting HTN = 2 as a function of each input feature value.

In DM prediction, both RF and GB models show similar trends in terms of PDPs for each input feature. Interestingly, it appears how a high mean asymmetry in the mean fat (∆μfat) as well as a high asymmetry in the heterogeneity of muscle (Δσmus) tissue composition contribute to increasing DM probability. These results complement observations from a previous study [4], which suggested that values of mean asymmetry of fat and muscle tissues, as measured from radiodensity distributions in mid-thigh CT cross-sections, are higher in subjects with high body-mass-index (BMI), which is a well-recognized risk-factor in type-2 DM [5]. Therefore, the classification algorithm was able to capture the significant relationships between the risk of developing DM and the unbalances in fat and muscle tissue composition (represented by ∆μfat and Δσmus).

In the following (Figures from S-2 to S-4) PDPs are illustrated and commented for each of the three classes of HTN prediction task.

Similarly to the case of binary DM prediction, in the HTN classification task, both RF and GB models show similar trends in terms of PDPs for each predictor. Even in the case of HTN, mean fat (∆μfat) and mean muscle (∆μmus) tissue asymmetry, along with asymmetry in tissue heterogeneity for fat (Δσfat) and muscle (Δσmus), proved to be factors potentially affecting the probability of transitioning from a no-hypertension status to a hypertension status. Indeed, the probability of being in the HTN = 0 class (i.e., no hypertension) decreases with ∆μfat and ∆μmus as well as with ∆σfat and ∆σmus (Figure S-2). Accordingly, probabilities of being in classes HTN = 1 and HTN = 2 increase with ∆μfat and ∆μmus as well as with ∆σfat and ∆σmus (Figures S-3 and S-4). Considering the association between BMI and soft tissue asymmetry (particularly evident in fat and muscle asymmetry), as observed in a previous study [4], and taking into account that BMI is a relevant predictor of hypertension conditions [6], the predictive models here presented provide results that are in line with the literature and are potentially able to identify the crucial role of fat and muscle asymmetry in predicting pathological conditions like DM and HTN.

Additionally, focusing on the model providing the highest accuracy in both classification tasks (i.e., the RF algorithm, see Table VII of the main manuscript), we calculated SHAP values and displayed them in a violin-like summary plot, which illustrates the global importance of features in predicting outcomes, as displayed in the following Figure S-5 (for binary DM prediction) and Figure S-6 (for three-classes HTN prediction).

In the latter case (Figure S-6), two SHAP summary plots are displayed for HTN = 0 (no hypertension) and HTN = 2 (hypertension) in order to compare the global feature importance and its effect on model prediction for two opposite classes (no-HTN status vs HTN status).

FIGURE S-1 Partial dependence plots (PDPs) for (A) Random Forest (RF) and (B) Gradient Boosting (GB) with the use of SMOTE for binary classification of Diabetes Mellitus (DM). Feature importance for this model is reported in Figure 4 of the main manuscript.

FIGURE S-2 Partial dependence plots (PDPs) for (A) Random Forest (RF) and (B) Gradient Boosting (GB) with the use of SMOTE for three-classes classification of Hypertension (HTN). Given the multiclass nature of the task, PDPs are here shown for the prediction of class HTN=0, i.e., subjects with no hypertension. Feature importance for this model is reported in Figure 5 of the main manuscript.

FIGURE S-3 Partial dependence plots (PDPs) for (A) Random Forest (RF) and (B) Gradient Boosting (GB) with the use of SMOTE for three-classes classification of Hypertension (HTN). Given the multiclass nature of the task, PDPs are here shown for the prediction of class HTN=1, i.e., subjects with pre-hypertension. Feature importance for this model is reported in Figure 5 of the main manuscript.

FIGURE S-4 Partial dependence plots (PDPs) for (A) Random Forest (RF) and (B) Gradient Boosting (GB) with the use of SMOTE for three-classes classification of Hypertension (HTN). Given the multiclass nature of the task, PDPs are here shown for the prediction of class HTN=2, i.e., subjects with hypertension. Feature importance for this model is reported in Figure 5 of the main manuscript.

FIGURE S-5 SHapley Additive exPlanations (SHAP) summary plots summary plot for the Random Forest (RF) with the use of SMOTE for binary classification of type-2 Diabetes Mellitus (DM).

In the above SHAP summary plot, insights into feature importance and their effects on model predictions are shown [2]. In particular, each feature value's impact on the model prediction is reported on the y-axis. The x-axis highlights the degree of changes in log-odds (logarithm of the odds values) of the predicted probability of being in the target class. The spread across x-axis reveals potential interactions or outliers, with highly impactful features with wide spreads indicating more complex interactions, whereas features with narrow spreads suggesting consistent effects on model prediction. The color bar indicates the feature value (from low to high). Features are sorted by their average impact, as reflected by their overall *mean(|SHAP value|)*, i.e., the mean absolute SHAP values across all instances, which represents how much, on average, the feature contributes to the model’s predictions regardless of whether the contribution is positive or negative.

In the case of DM prediction (Figure S-5), the role of fat tissue is the most evident. Indeed, it can be seen how higher values in fat asymmetries (ΔNfat, Δσfat) have a high and consistent impact on the model output, thus increasing the log-odds of being in the DM = 1 class. More complex interactions (wider spreads) on model predictions are instead observed for muscle and connective tissue asymmetries.

FIGURE S-6 SHapley Additive exPlanations (SHAP) summary plots for Random Forest (RF) with the use of SMOTE for three-classes classification of Hypertension (HTN). Given the multiclass nature of the task, SHAP summary plots are here shown for the prediction of class (A) HTN=0, i.e., subjects without hypertension, and (B) HTN=2, i.e., subjects with hypertension.

In the case of HTN prediction, wider spreads are observable for HTN = 0 class (Figure S-6 A), denoting complex feature patterns in determining the prediction of no-hypertension conditions. Conversely, narrower spreads, with clearer roles played by both fat (ΔNfat, ∆μfat, Δαfat) and muscle (Δσmus) tissue asymmetries, are shown in the case of hyppertension conditions (Figure S-6 B), confirming how high values for the mentioned asymmetry indicators express increased log-odds of being in the HTN = 2 class.

References

[1]	A. Mohanty and S. Mishra, “A Comprehensive Study of Explainable Artificial Intelligence in Healthcare,” Augmented Intelligence in Healthcare: A Pragmatic and Integrated Analysis, pp. 475–502, 2022

[2]	N. Gandhi and S. Mishra, “Explainable AI for Healthcare: A Study for Interpreting Diabetes Prediction,” Lecture notes in networks and systems, pp. 95–105, Sep. 2021, doi: https://doi.org/10.1007/978-3-030-82469-3_9.

[3]	Y. Nohara, K. Matsumoto, H. Soejima, and N. Nakashima, “Explanation of machine learning models using shapley additive explanation and application for real data in hospital,” Computer Methods and Programs in Biomedicine, vol. 214, p. 106584, Feb. 2022, doi: <https://doi.org/10.1016/j.cmpb.2021.106584>.

[4]	C. Ricciardi et al., “Development of soft tissue asymmetry indicators to characterize aging and functional mobility,” Frontiers in bioengineering and biotechnology, vol. 11, Dec. 2023

[5]	N. Gray, G. Picone, F. Sloan, and A. Yashkin, “Relation between BMI and Diabetes Mellitus and Its Complications among US Older Adults,” Southern Medical Journal, vol. 108, no. 1, pp. 29–36, Jan. 2015, doi: https://doi.org/10.14423/smj.0000000000000214.

[6]	W. Qiao et al., “Hypertension, BMI, and cardiovascular and cerebrovascular diseases,” Open Medicine, vol. 16, no. 1, pp. 149–155, Jan. 2021, doi: https://doi.org/10.1515/med-2021-0014.
